# Editorial and peer review dynamics at elite general science journals

**DOI:** 10.1126/sciadv.aec0494

**Published:** 2026-07-15

**Authors:** Sam Zhang, Nicholas LaBerge, Quinten McElhiney, Daniel B. Larremore, Aaron Clauset

**Affiliations:** ^1^ Department of Mathematics and Statistics, University of Vermont, Burlington, VT, USA.; ^2^ Santa Fe Institute, Santa Fe, NM, USA.; ^3^ Applied Mathematics Department, University of Colorado, Boulder, CO, USA.; ^4^ Computer Science Department, University of Colorado, Boulder, CO, USA.; ^5^ BioFrontiers Institute, University of Colorado, Boulder, CO, USA.

## Abstract

Elite general science journals shape scientific discourse, public policy, and scientific careers. However, expectations of confidentiality in most editorial proceedings has limited efforts to understand and improve the review process. Here, we describe and release deidentified data on 110,303 manuscript submissions over 5 years at *Science* and *Science Advances*, two elite general science journals. Analyzing evaluation dynamics across the initial editorial review and subsequent peer review stages, we find strong selective effects associated with higher institutional prestige, larger team size, and certain topics and countries. Corresponding authors who are men exhibit a small but significant advantage at *Science*, while authors based in China have a significant disadvantage. These associations are generally stronger in editorial review than in peer review, even as final editorial decisions correlate strongly with reviewer advice. These patterns highlight the complexity of multistage evaluations at elite journals and the importance of open data to better understand them.

## Teaser

Analyses of deidentified data reveal complex interactions that quantify success at elite general science journals.

## INTRODUCTION

Peer review by scientific experts has become the gold standard for critically assessing whether a completed study meets or surpasses certain standards for the scientific record. Ideally, peer review is reliable (*1*, *2*), timely (*3*), and correct (*4*, *5*) and minimizes bias (*6*).

The stakes of peer review are especially high at elite general science journals, whose published articles tend to receive more public scrutiny, exert more scientific and policy influence, and weigh more heavily in decisions about tenure and promotion, grant funding, and prestigious awards (*7*, *8*). As a result, peer review at elite general science journals disproportionately shapes the direction and pace of scientific discovery (*4*, *7*, *9*), the arc of individual careers, and the composition of the scientific community (*8*, *10*). However, elite journals receive a far greater volume of submissions, limiting how much prior studies of peer review can generalize to the most prominent venues. Moreover, elite journals typically operate using single-blind review, and the expectation of confidentiality severely limits the availability of data for scientific studies of peer review itself, e.g., data on the pool of submissions, author and manuscript characteristics, editor evaluations, reviewer selections, peer reviews, and final decisions. This lack of peer review data at elite, general science journals, in turn, makes it difficult to assess how well they achieve the scientific community’s ideals, or to develop and test theories to improve the process.

Here, we make two specific contributions. First, we introduce a detailed dataset of the evaluations of 110,303 manuscripts submitted between 2015 and 2020 to *Science* and *Science Advances*, two elite multidisciplinary journals. We standardized and augmented these data with manuscript-level covariates, and we provide, with permission, a deidentified version for public use (see the “Data, code, and materials availability” section in Acknowledgments). Second, we describe a sequence of statistical analyses that quantify the evaluation dynamics of submissions to these journals, shedding new light on the way editors and experts shape the outcomes of peer review.

Because there is no objective measure of a manuscript’s quality that is ex ante independent of an evaluation by experts, editors at elite journals face a substantial challenge: the sheer volume of submissions. In 2023, *Science* received more than 10,000 submissions but published just 6.1%, which is similar to *Nature*’s rate of 8% and to other elite journals without a general science focus: 6.3% at the *American Sociological Review*, 5% at the *New England Journal of Medicine*, and 5% at the *American Economic Review* (*11*–*14*).

To manage high submission volumes and maintain high standards for published articles, elite journals today (and many other journals, to a lesser extent) typically evaluate manuscripts in a two-stage process: editorial review, where journal editors screen and select a smaller set of promising manuscripts, and peer review, where editors recruit outside experts to carefully vet and suggest improvements to selected submissions, before making a final decision to accept or reject the manuscript (see section S1). Although decisions are ultimately made by the editor in both stages, we use the terms editorial review and peer review as mutually exclusive labels that distinguish the two separate stages.

Research on manuscript evaluation has typically focused on less selective journals that serve specific fields of science, centering the peer review step rather than the work of the editor. At elite journals, editors play a central role, rejecting a large majority of submissions without formal peer review, recruiting specific outside experts as reviewers, and guiding authors in how to respond to them. Hence, a full understanding of reliability (*1*) and social biases (*6*) in “peer review” requires an integrated study that spans editorial and peer review stages. Despite the importance for the scientific community of examining and improving these processes at elite general science journals (*15*), the kind of detailed data needed for integrated analyses is sharply limited (*16*) by journal commitments to protect reviewer confidentiality.

As a result, very few studies have generated quantitative insights into the evaluation processes in elite general science journals. *Science* began as a privately owned journal in 1880, and became the official journal of the American Association for the Advancement of Science (AAAS) in 1900. At the time, it employed professional editors who made the editorial decisions themselves without the consistent assistance of peer reviews. *Science* only began relying on outside experts as reviewers in the 1950s, after the editors complained of the unpleasant burden of conducting hundreds of technical peer reviews themselves (*17*). In 1985, *Science* introduced an editorial review stage by creating the “Board of Reviewing Editors” (BoRE), a set of outside experts who would provide rapid advice to the editors on whether and to whom to send a manuscript for formal peer review. Editorial review decreased the burden faced by both editors and peer reviewers in sorting the large volume of submissions, and allowed *Science* to provide faster feedback to authors in most cases (*18*). Throughout its entire history, *Science* has employed professional editors.

*Science Advances* started as the open-access sibling journal to *Science* in 2015, covering all the same areas of science with similarly high standards, but run by academic editors who conduct the editorial review step and then oversee formal peer review (see section S1). The differences between the two journals allow us to examine whether structural differences lead to observable differences in editorial outcomes.

In studies of peer review, causal evidence is particularly important, with anonymization experiments representing an ideal standard for identifying bias (*6*, *19*–*23*). However, releasing open datasets and characterizing disparities in outcomes at elite journals is valuable in its own right (*15*), in much the same way that evidence of gender pay gaps in certain occupations (*24*) or racial discrimination in policing (*25*) is relevant to society even without clear causal mechanisms at first. The detailed dataset we make available and the statistical results we describe below shed new light on how elite general science journals evaluate scientific contributions, and help focus attention on how to improve them.

## RESULTS

### Deidentified data on peer review

From a complete set of manuscript records (2015–2020) provided by AAAS, which publishes *Science* and *Science Advances*, we clustered all regular article submissions into 10 broad topic areas based on their titles and abstracts, and annotated each with their editorial decisions, number of authors (team size), first and corresponding author characteristics (institutional prestige, gender, and geographic region), BoRE evaluations with score (1 to 10) and confidence (if applicable), and review characteristics (length and algorithmically estimated sentiment, as *z*-scores, a reviewer evaluation selected from one of five categorical choices, a sentiment “trajectory” label that characterizes the rise and fall of reviewer sentiment over the course of the review, and reviewer gender and institutional prestige). To preserve anonymity, personally identifiable and reidentifiable information, such as the text of the reviews, was removed, and only characteristics of two randomly chosen first-round peer reviews were included for each reviewed manuscript. The resulting deidentified dataset is provided, with permission, to the research community for analysis and reuse (see section S2).

**Fig. 1.**
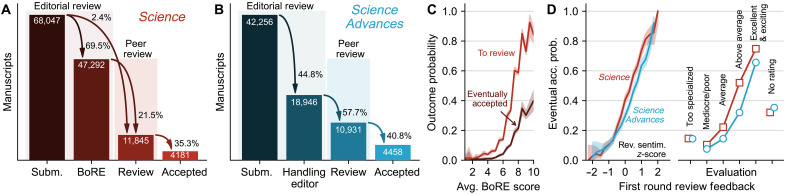
Editorial processes at *Science* and *Science Advances*. (**A** and **B**) Scientific manuscript counts and progression rates for editorial review and peer review stages at *Science* (red, all panels) and *Science Advances* (blue, all panels), 2015–2020. (**C**) Rates of progression to peer review and eventual acceptance based on BoRE scores at *Science*, and (**D**) rates of eventual acceptance versus average first round review sentiment *z*-score (plotted only for points with 95% CI widths under 15%) and categorical reviewer feedback. (Abbreviations: Subm., submitted; Avg. BoRE score, Average Board of Reviewing Editors Score; Rev. sentim., review sentiment; acc. prob., acceptance probability.)

Over the study period, the overall acceptance rate of *Science* was 6.1% and that of *Science Advances* was 10.5%. Both journals use the two-stage evaluation process of editorial review followed by peer review on a subset of submissions. Editors at *Science* summarily rejected 28.1% of the 68,047 submissions manuscripts and sent 69.5% to external experts on the BoRE. Given BoRE ratings and advice, editors declined 75% of those manuscripts, sending the remainder to peer review (Fig. 1A).

Among reviewed manuscripts, acceptance rates were 35.3% at *Science* and 40.8% at *Science Advances*, making conditional success in the peer review stage markedly higher than the corresponding rates of 17.4% and 25.9% over the editorial review stage (Fig. 1, A and B). Although manuscript quality is an unobserved confound in all peer review data, the higher conditional success rate in peer review versus editorial review is consistent with the central role of editors and prereview advisors in selecting a subset of manuscripts that are more likely to pass rigorous expert evaluation. However, we lack counterfactual data on how reviewers would evaluate editorially rejected manuscripts.

Nevertheless, advice from external advisors and peer reviewers correlates strongly with editorial outcomes. During editorial review, manuscripts at *Science* receiving BoRE scores of 8 to 10 went to review 82% of the time, compared with 5% for scores of 1 to 5 (Fig. 1C). During peer review, manuscripts with an average first-round review sentiment 1 SD below the mean (*z* = −1) were eventually accepted just 10% of the time, compared to 72% for 1 SD above the mean (*z* = 1; Fig. 1D), with similar patterns for reviewer evaluations at *Science Advances*.

**Fig. 2.**
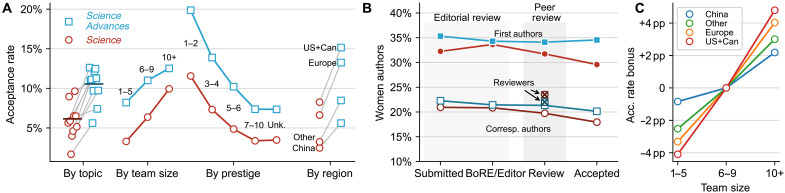
Correlates of success at *Science* and *Science Advances*. (**A**) Overall acceptance rates at *Science* (red circles) and *Science Advances* (blue squares) by topic, team size (number of authors), prestige groups (1 to 2, highest; 7 to 10, lowest), and corresponding author geographic region. Gray lines connect the same group across journals. (**B**) Women’s representation among first authors (solid symbols), corresponding authors (open symbols), and among first-round peer reviewers (crossed symbols) over the evaluation process. (**C**) Difference in acceptance rates between six and nine author teams versus other team sizes, stratified by corresponding author region. (Abbreviations: Unk., unknown; Can., Canada; BoRE, Board of Reviewing Editors; pp, percentage points.)

**Fig. 3.**
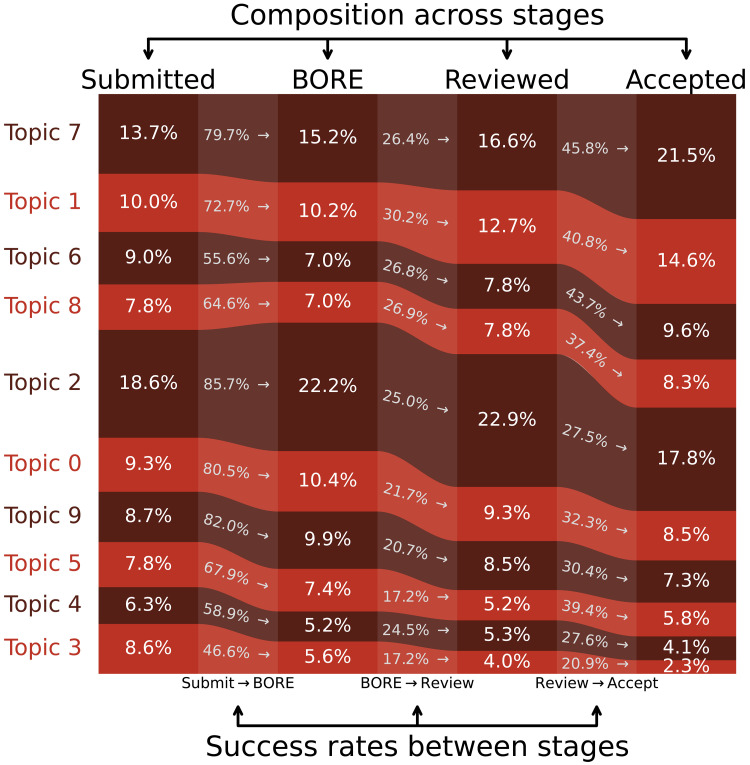
Topic representation across editorial stages at *Science*. Ten broad and deidentified topics are illustrated by their proportions among submitted, BoRE reviewed, peer reviewed, and accepted papers (white text) with success rates between stages calculated for reference (gray text). Topics are plotted in order from highest overall acceptance rate (topic 7) to lowest overall rate (topic 3). For a list of distinctive terms that characterize each topic, see table S13. For *Science Advances*, see fig. S1.

Within these overall rates is substantial variation in success by manuscript topic, and the prestige, team size, and region of the authors (Fig. 2A). For instance, 9.6% of topic 7 submissions—which includes research related to viruses, infection, RNA, and immune response (table S13)—were published in *Science*, compared to just 1.7% for topic 3, whose submissions concern topics including politics, economics, gender, and the societal dimensions of COVID (table S13). This nearly sixfold difference in acceptance rate reflects compounding disparities in how often submissions from each topic were forwarded to the BoRE (79.7% versus 46.6%), sent for external review (26.4% versus 17.2%), and ultimately accepted after review (45.8% versus 20.9%, Fig. 3). Manuscripts from the most prestigious quintile of corresponding authors had a 11.6% acceptance rate, compared to just 3.4% for the two least prestigious quintiles—a 3.4-fold difference. Papers from large teams were accepted at 3 times the rate of small teams (10+ authors, 9.9%; versus 1 to 5 authors, 3.3%), and papers with US- or Canada-based corresponding authors were accepted at 3.3 times the rate of China-based authors (8.3% versus 2.5%, respectively). Comparing *Science* and *Science Advances*, we find a qualitatively similar pattern of variation in acceptance rates with author prestige, team size, and geographic region (Fig. 2).

Acceptance rates at both journals also varied by author gender, but much less so than other manuscript-level covariates. Over editorial review and peer review stages, submissions to *Science* with men as corresponding authors fared marginally better than those with women, leading to a 1.1 pp (percentage points) difference in total acceptance rates (6.4% versus 5.3%, *P* < 0.001). Consequently, women’s representation among submitting authors was generally higher than among accepted authors, except for first authors at *Science Advances* where the 2.4% difference was not significant (*P* = 0.13; Fig. 2B).

### Correlates of manuscript success

Measuring the importance of a manuscript’s characteristics in explaining observed differences in success rates requires accounting for two complexities. First, manuscript characteristics are correlated. For example, author gender correlates with manuscript topic, and this correlation itself can account for 13% of the gendered difference in acceptance rates at *Science*. Similarly, topic can explain 9% of the difference in acceptance rates between the smallest and largest teams (see section S3). The apparent large-team advantage (or, small-team penalty) is twice as large for US-, Canada-, and Europe-based authors compared with that of China-based authors (Fig. 2C).

**Fig. 4.**
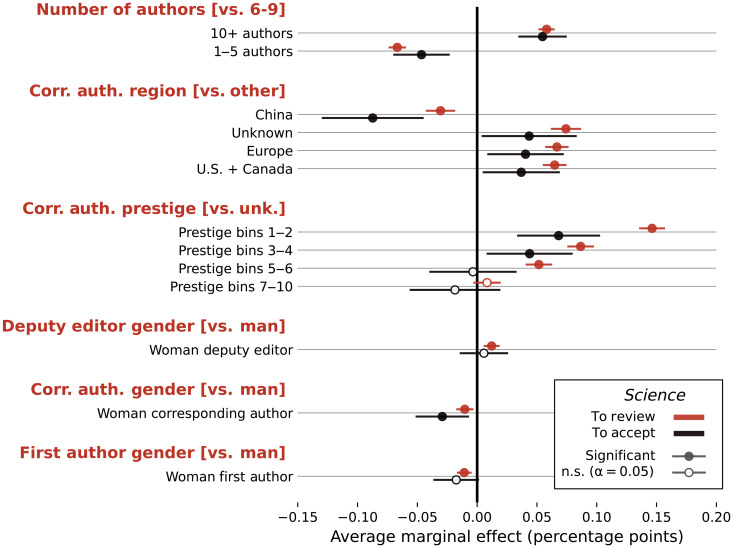
Manuscript characteristics and review stage success. Average marginal effects for each nontopic manuscript covariate at *Science*, controlling for topic: the average change in predicted probability (in percentage points) of (red) progressing from editorial review to peer review and (black) being accepted for publication after peer review, for any given covariate compared to the reference category. Reference categories are shown in brackets in each covariate label; statistical significance is indicated with a solid marker (*z* test, α = 0.05). Error bars indicate 95% CIs. *Science Advances* shows similar results (fig. S4).

Second, acceptance depends on success in editorial review and then again in peer review, which are both decided by the editor, meaning that total variation in success is a mixture of direct effects from the editor and indirect effects from advice to the editor. Modeling editorial review outcome at *Science* (is a manuscript sent for review?) as a function of observable author and manuscript characteristics, we find strong correlations with institutional prestige, geographic region, and team size (Fig. 4 and table S8; see Materials and Methods). Net of other covariates, the advantage of manuscripts from the most elite institutions (prestige bins 1 and 2) is nearly twice as large as that of middle-prestige institutions (bins 3 and 4), while the disadvantage of manuscripts with small teams (1 to 5 authors) is about as large in magnitude as the advantage of large teams (10+ authors), and geographically, only manuscripts with authors based in China exhibit a net disadvantage. In contrast, the gender of the handling editor, corresponding author, and first author exhibits only modest associations, and all are substantially smaller in magnitude than the estimated coefficients for prestige, team size, or geography (see section S3 for additional supporting analyses).

Modeling peer review outcome at *Science* (is a reviewed manuscript accepted?) with logistic regression, we find smaller associations across the board compared to editorial review (Fig. 4 and table S11). Such a shift is consistent with a screening effect for peer review, in which author and manuscript characteristics become less salient, conditioned on also having the perspective of outside experts (see Materials and Methods). The advantage for manuscripts from the most prestigious institutions (prestige bins 1 and 2) has the greatest change, falling to about one-third its size in the final manuscript decision compared with initial editorial review. Associations with handling editor gender, corresponding author gender, and first author gender remain modest or insignificant. We note that the disadvantage of manuscripts with a China-based corresponding author does not change significantly across editorial and peer review stages (*z* = 1.536, *P* = 0.124). We find similar patterns at *Science Advances*, but with some minor differences (see fig. S4).

To assess the relative degree to which these manuscript-level patterns may arise from the editors themselves versus the advice of outside experts, we performed a series of mediation analyses using BoRE ratings for editorial review and review sentiment scores for peer review to isolate the indirect effects of these advisors on outcomes (see section S4 for further discussion of the assumptions in the analysis).

**Fig. 5.**
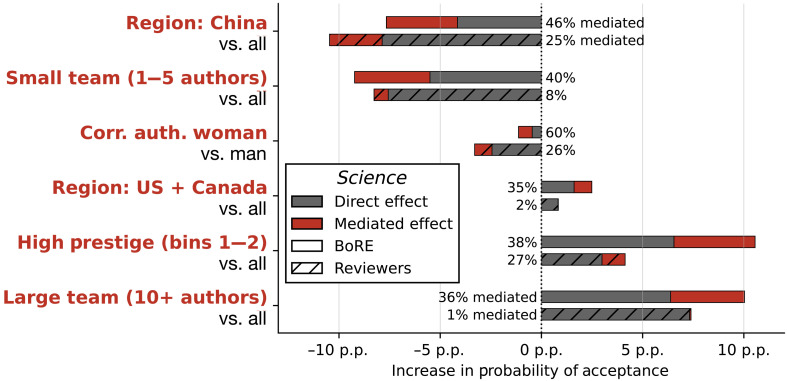
Expert mediation of editor decisions. Decomposition of the increase in probability of acceptance, controlling for available covariates, into the portion attributable directly to the editor (gray) versus mediated by the expert (red; percentage annotations) for selected covariates in the editorial and peer review stages at *Science*. The BoRE ratings mediate the editor’s decision in editorial review (solid), and the review sentiments mediate the editor’s final decision in peer review (striped). *Science Advances* shows similar results (fig. S6).

During editorial review at *Science*, BoRE advisors and editors are responsible for similar proportions of the observed associations of author and manuscript characteristics with success (Fig. 5, fig. S6A, and table S2). We estimate that the BoRE accounts for 37.9% of the prestige association, 41.7 to 45.8% of the author geographic region association, 36.4 to 40.3% of the team size association, and 61.2% of the already marginal gender association. A similar pattern of split responsibility appears in peer review at *Science*, but here, editors account for a larger portion of the observed author and manuscript associations (at least 66.9% for any covariate) compared to the reviewer (table S3), with qualitatively similar patterns appearing for peer review at *Science Advances* (table S3). The larger role of editors compared to reviewers in explaining the correlates of success in the peer review stage is consistent with an attentional hypothesis, in which outside experts apply greater subject matter expertise in evaluating manuscripts than do editors, which reduces the influence of author and manuscript attributes (*26*). Alternatively, editors may have distinct preferences, e.g., to select for manuscripts with broad appeal to a multidisciplinary audience, compared to reviewers, who may be more focused on technical aspects of the manuscript.

### Gendered effects in peer review

At *Science* and *Science Advances*, manuscripts with women corresponding authors make up a gradually declining share from submission to review to acceptance (20.9%→20.8%→19.7%→17.9% and 22.3%→21.4%→21.3%→20.1%, respectively; Fig. 2B). Accounting for available covariates via multiple logistic regression reveals a small but statistically significant association with corresponding author gender in being sent to review or accepted after review (β = −0.08, *P* < 0.001 for sent to review; β = −0.13, *P* = 0.011 for accepted; tables S8 and S11).

Although the associations with corresponding author gender are small compared to other covariates (e.g., the average causal mediation effect of prestige on BoRE ratings is 0.04 compared to −0.007 for corresponding author gender; see table S2), their cumulative association over editorial and peer review appears meaningful in terms of outcomes, e.g., in units of publications. While these data do not support definitive causal claims (see Discussion), it is nevertheless useful to consider that under a counterfactual model in which all manuscripts with women corresponding authors are assigned the men corresponding author coefficient, we estimate that *Science* could potentially have published 18.4 ± 4.9 (mean ± SD) additional articles per year with women corresponding authors, about a 12 ± 3% increase (see section S5, and table S4).

In contrast, first author gender exhibits no consistent association in any stage of the review process, except that women first authors comprise a slightly larger share of desk rejections at *Science*, but not *Science Advances* (β = −0.08, *P* < 0.001 for *Science*; β = −0.02, *P* = 0.40 for *Science Advances*; table S8). At each stage, the share of women as first authors is, on average, 13.4 pp greater than the share of corresponding authors.

Theoretical work on peer review suggests four potential mechanisms by which peer reviewers may bias their evaluations according to perceived author gender: (H1) gender homophily, where reviewers favor authors of the same gender (*27*, *28*); (H2) men favor other men, while women are gender neutral; (H3) women favor men, while men are gender neutral; and (H4) all evaluators favor men (*29*).

We evaluate the evidence for each of these mechanisms by comparing the average sentiment that authors of one gender receive from reviewers of the same or different gender, e.g., if MW is the mean sentiment of reviews by women reviewers for manuscripts with a man as corresponding author, then MW > MM indicates that, on average, men authors receive reviews with more positive sentiment from women reviewers.

**Fig. 6.**
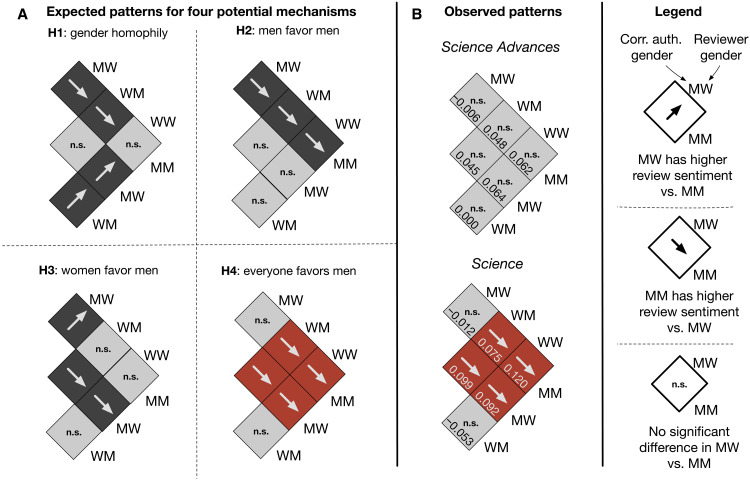
Gendered peer reviews. (**A**) Expected patterns of gendered review sentiment under four potential mechanisms: H1: gender homophily, in which men reviewers M favor men authors and women reviewers W favor women authors; H2: men reviewers favor men authors, while women favor neither; H3: women reviewers favor men authors, while men favor neither; and H4: both women and men reviewers favor men authors. Each mechanism makes a distinct set of predictions over the six possible comparisons of pairings of corresponding author and reviewer binary gender. An arrow points to the combination with the greater relative sentiment, e.g., H1 predicts MW → MM and WW ← MW. (**B**) Observed patterns for *Science* and *Science Advances*, using propensity score weighting to control for covariate differences between the comparison groups. Statistical significance by *t* test, with Bonferroni correction *P* < 0.0083.

The four mechanisms make a distinct patterns of predictions for the six possible binary pairings of corresponding author and reviewer gender (Fig. 6A), allowing us to test them in parallel with appropriate corrections for multiple comparisons. Using propensity score weighting to control for covariate differences between comparison groups (see section S6), at *Science*, we find that both men and women reviewers produce modest but statistically significantly more positive reviews to teams with men as corresponding authors (H4; Fig. 6B, Bonferroni corrected *P* < 0.0083). In contrast, we find no significant favoring among any gender pairs at *Science Advances*. We note that these results do not necessarily indicate a gender bias among peer reviewers at *Science*. One alternative explanation of the observed advantage for men at *Science* could be unmodeled seniority effects.

We observe large negative associations with success for manuscripts with China-based corresponding authors. Using the underlying nonanonymized data to compare success rates of (i) corresponding authors with Chinese last names based in China versus those based in the United States, and (ii) corresponding authors based in the United States with Chinese versus non-Chinese last names, we find statistically significant correlations between both Chinese last names and Chinese affiliations with negative manuscript outcomes at nearly every step of the review process at both journals (see section S7 and fig. S8).

## DISCUSSION

How do the editorial processes of elite general science journals shape the epistemic landscape of science? The broad lack of data from elite journals makes it difficult to answer such a question scientifically, and as a result, peer review at elite journals is both undertheorized and undercharacterized, despite many insights from past studies about peer review in general. The deidentified large-scale data we introduce here provides an unusually rich set of individual and manuscript-level variables at each stage of the evaluation process at two elite journals, and our analyses of that data reveal the complexity of their editorial and peer review processes.

Across editorial and peer review at *Science* and *Science Advances*, editorial review emerges as the much stronger filter, as the likelihood of success in editorial review is substantially lower than the subsequent likelihood of success in peer review. In this way, editorial review may increase the underlying quality of manuscripts that reviewers assess, compared to a model where all submissions are externally peer reviewed. A stringent editorial review stage also allows editors to shape the topic distribution of published articles (Fig. 3; see section S2.4). This model of editorial review markedly reduces the number of reviewers that need to be recruited, allocates the attention of outside experts more efficiently, and provides rapid feedback to most authors, despite enormous volumes of submissions. Because peer review as a service is generally underprovisioned, stronger editorial filtering may be beneficial for the entire scientific community (*30*).

At the same time, stringent editorial review exhibits more correlation with author and manuscript characteristics, which have uncertain relationships with underlying manuscript quality. For instance, institutional prestige, manuscript topic, geographic location, and team size exhibit stronger net associations with success in editorial review than in the peer review (Figs. 4 and 5), although these associations also appear for peer reviews.

This step down in association strength from editorial to peer review is consistent with two different hypotheses: one statistical and one attentional. The statistical explanation is that because peer review occurs on the subset of submissions that passed editorial review, we cannot determine the extent to which correlations between observed demographics and outcomes may be due to conditioning on a collider (see section S4 and fig. S7). The attentional hypothesis states that reviewers are less sensitive than editors to author characteristics and more narrowly focused on the manuscript’s underlying quality (*26*), although it is also likely that editors at elite journals use broader evaluation criteria than outside reviewers. These patterns span both *Science* and *Science Advances* despite the fact that *Science* employs full-time editors and *Science Advances* relies on academic editors (see section S1).

Manuscript topic is among the most important manuscript characteristics for its success in both editorial and peer review (Figs. 2A and 3), with acceptance rates ranging from 1.7 to 9.6% across the 10 high-level topics at *Science*. Variation in success by topic may reflect underlying differences in quality across topics or editorial preferences or targets on topics, e.g., an oversupply of submissions on artificial intelligence. The advantage for large teams may be due to a greater reputational risk to the journal from rejecting the work of many experts, or may reflect an editorial preference for the type of epistemic advance that requires a large team to produce (*31*), e.g., forecasting plastic pollution (*32*) and the Human Genome Project (*33*). The statistically marginal effect of corresponding author gender on editorial and peer review success, and on review sentiment at *Science* (Fig. 6B) may reflect a slight systematic bias against women in these high-stakes assessments, e.g., due to stereotype effects (*34*). However, the lack of parallel patterns between first and corresponding author gender, or at *Science Advances*, suggests that the gender associations may reflect unmodeled seniority effects, as current demographics make men statistically more likely to be senior scholars (*35*, *36*).

In general, bias is difficult to identify separately from quality from these data. For instance, the prestige and team size associations likely reflect a combination of status signaling effects and resource advantages among elite institutions and larger teams that may independently improve manuscript quality. Large negative associations with success for manuscripts with China-based corresponding authors are notable. The high volume of such submissions across topics may reflect a region-specific incentive to submit work to high-impact journals regardless of suitability, such as China’s past publication bounty program (*37*). Editors then rebalance the submission pool to filter these out during editorial review.

Of the available author and manuscript characteristics, associations for prestige and topic with evaluation success are enormous, e.g., compared to that of author gender, even as author gender is among the most broadly studied manuscript characteristic in peer review (*6*). The magnitude of these associations raises an important question: To what extent do the editors and reviewers at elite journals tend to overlook great scientific contributions that lack these advantageous author and manuscript characteristics? Past work suggests that interdisciplinary research and particularly novel ideas can be undervalued in peer review (*38*), but it remains unclear how well they fare under editorial review at elite journals, which may have an incentive to favor such work.

Preserving confidentiality in the deidentified dataset required summarizing the text of peer reviews via simple measures like length and sentiment. While review sentiment does strongly correlate with peer review outcomes (Fig. 1D), deeper insights could be drawn from analyzing the rhetorical structure of reviews and author responses, which are not included in the deidentified dataset. Models of how reviewers and authors interact would extend our understanding of the priorities placed on gatekeeping, correctness, or improvement within peer review at elite journals, and while the topics we defined are data driven, their coarseness is certainly obscuring more fine-grained preferences among editors and reviewers, as well as interdisciplinary aspects of articles.

Our analyses illustrate the potential utility of the deidentified dataset for future studies of elite peer review analysis. For instance, the deidentified dataset includes a number of covariates that were not specifically analyzed here, and there are novel analyses that could be performed on these and other variables. For example, it could be used to investigate the role of geographic homophily between authors and reviewers (*39*), or it could be used to investigate the role of reviewer prestige in peer review, which is understudied relative to the role of author prestige (*20*, *40*). More detailed interactions could be studied between the characteristics of the first or corresponding author and reviewer, as well as between the authors and the editor. These data also open the possibility of carefully delineating the role of BoRE, editor, and reviewer gender on manuscript evaluations.

Identifying the mechanisms that drive the observed associations, and the extent to which they are due to biases from editors or reviewers (*6*, *23*), will require new efforts with experimentation (*20*, *40*). The two-stage structure of evaluations at elite journals presents multiple opportunities to intervene, e.g., anonymizing author characteristics in either or both stages. Statistical power estimates suggest that some effects may be feasibly identified through anonymization experiments; e.g., even if the true influence of prestige, country, or team size on BoRE ratings were only half their estimated regression coefficients (table S7), we could detect statistically significant effects by anonymizing about a third of the submissions to *Science* in a year (section S8).

Detailed data on and experiments with editorial and peer review are an essential part of critically examining the processes that we, the scientific community, use to advance scientific knowledge. As a structured social process for managing debate among experts, there are many opportunities for nonmeritocratic factors to influence editorial and peer review outcomes. It is imperative that we apply scientific principles to investigate how different factors and their interactions influence the success of different types of manuscripts in the high-stakes evaluations at elite general science journals.

## MATERIALS AND METHODS

Editorial review outcome (whether a manuscript is sent for review) and peer review outcomes (whether a reviewed manuscript is accepted for publication) are modeled using logistic regression. Both models include the same covariates

$$\begin{array}{c} \text{logit}\left[P\left(Y=1\right)\right]=\beta_{0}+\beta_{\#\text{authors}}+\beta_{\text{Corr}.\;\text{auth. region}}+\beta_{\text{Corr}.\;\text{auth. prestige}}\\ +\beta_{\text{Deputy editor gender}}+\beta_{\text{Corr}.\;\text{auth}.\;\text{gender}}+\beta_{\text{First auth}.\;\text{gender}}+\beta_{\text{Topic}}+\varepsilon \end{array}$$

Results with alternative model specifications are reported in the Supplementary Text, such as including BoRE ratings for editorial review (table S9) and reviewer evaluations and sentiment for final acceptance (table S12).

## Supplementary Materials

### This PDF file includes:

Supplementary Text Tables S1 to S13 Figs. S1 to S8 References
