# Supplementary material for: Editorial and peer review dynamics at elite general science journals
Source: Sci Adv. 2026 Jul 15;12(29):eaec0494. doi: 10.1126/sciadv.aec0494 (PMC13371883; doi:10.1126/sciadv.aec0494)
Supplement: Supplementary file 1 — Supplementary Text Tables S1 to S13 Figs. S1 to S8 References [file sciadv.aec0494_sm.pdf]

Science Advances
AAAS

Supplementary Materials for

Editorial and peer review dynamics at elite general science journals

Sam Zhang *et al.*

Corresponding author: Aaron Clauset, aaron.clauset@colorado.edu

Sci. Adv. **12**, eaec0494 (2026)
DOI: 10.1126/sciadv.aec0494

This PDF file includes:

Supplementary Text
Tables S1 to S13
Figs. S1 to S8
References

Appendix S1: Overview of Editorial and Peer Review at *Science* and *Science Advances*

The evaluation of manuscripts at *Science* and *Science Advances* can be divided into two complementary stages: editorial review and peer review. Both stages are managed by journal-specific, non-overlapping groups of editors, i.e., editors at *Science Advances* are editorially independent of editors at *Science*.

Editors at *Science* are "professional," meaning they are full-time employees of the journal. All editors at *Science Advances* are "academic," meaning they hold full-time academic appointments elsewhere, and act as editors for the journal on a part-time basis. Both journals are broad and multidisciplinary, nominally publishing in every area of science. Hence, both journals' editorial boards and reviewer pools are multi-disciplinary.

The editorial review stage at each journal is slightly different, due in part to the differences in their editorial boards. A rejection at the editorial review stage is often called a "desk rejection."

At *Science*, editors summarily reject 30.5% of submissions, and the remaining 69.5% are sent to one or more members of the Board of Reviewing Editors (BoRE), a set of about 400 scientific experts with active academic appointments elsewhere. The BoRE provide rapid expert assessments of these submissions, in the form of a score 1–10, a confidence 1–5, and, optionally, comments and recommendations for potential reviewers. Based on these rapid assessments, *Science* editors reject 75% of these manuscripts without further review (Fig. 1A). The remaining manuscripts move on to the peer review stage.

At *Science Advances*, manuscripts are initially evaluated by a deputy editor (DE) with relevant scientific expertise, based on the manuscript's topic. On the basis of this initial evaluation, DEs reject 55.2% of manuscript (Fig. 1B). The DE then assigns the manuscript either to themselves or to an (academic) associate editor (AE) with relevant scientific expertise to act as the "handling editor" to manage its evaluation in the peer review stage. However, at *Science Advances*, AEs also perform an initial evaluation, and only 57.7% of manuscripts assigned to an AE move on to the peer review stage.

Authors whose manuscripts are rejected at *Science*, either during editorial or peer review, may elect to transfer their manuscript to be considered by *Science Advances*. Out of 45,951 submission to *Science Advances*, 12,537 (27.2%) were transferred from *Science*. If a manuscript was sent for review at *Science*, authors can choose whether to transfer it with or without those reviews (which the authors see prior to deciding). Statistically, a manuscript transferred without reviews is most likely to have been desk rejected at *Science*. However, it could also be a manuscript that was reviewed at *Science* but whose authors did not want the *Science* reviews to be seen by the *Science Advances* editors. To the *Science Advances* editors, both such cases appear the same to them: a transfer without reviews. Of the transferred manuscripts, only 702 (5.6% of transfers, or 1.5% of all *Science Advances* submissions) were transferred with reviews. Of those manuscripts, 353 (50.3%) received further reviews, and only 215 (30.6%) were ultimately accepted—a lower rate than other reviewed manuscripts at both *Science* and *Science Advances* (Fig. 1A). For our analyses, we ignore the small number of reviews transferred from *Science* for our analyses, and consider them, as well as all other transfers, as de novo submissions.

At *Science*, each editor is given a target "page budget" for the number of articles they can publish each year, which is approximately equal to $1/k \times 750$ papers, where k is the number of editors (up to historical trends and opportunities). In this way, the distribution of editors over topics shapes the overall proportion of papers *Science* publishes in different topics, because some high-level areas have many editors, while others have only a few. In contrast, *Science Advances* is an online-only journal (compared to *Science* being a print journal during the period of study). Hence, *Science Advances* editors have no page budgets or similar constraints on the number of papers they can publish in a year, and editors are instructed to consider all papers of sufficient quality and broad interest.

The peer review stage at both journals has the same structure: the "handling editor" recruits individual experts to submit reviews of a particular submission, evaluates the reviews and manuscript together, and then decides to either accept, reject, or send the manuscript back to the authors to be revised and resubmitted for another "round" of peer review. Both journals require a minimum of two reviews to accept a paper, but a paper may be rejected with any number of reviews (including zero, a "desk rejection" during editorial review). It is up to the editor to decide when and under what conditions a round of review is complete and a decision (accept, reject, revise) is made. Over the study period, 72.2% of reviewers submitted exactly 1 review to either journal, and the mean number of reviews by any reviewer was 1.56.

The reviewer pools used by both journals are broad and only partially overlapping. Over our study period, 71% of reviewers at *Science*, and 82% of reviewers at *Science Advances*, reviewed only once. However, reviewing exhibits a long tail of activity, with some reviewers offering advice on many manuscripts. At *Science*, the 43 people (0.20%) who reviewed 10 or more manuscripts account for 2.2% of all reviews. Similarly, at *Science Advances*, the 41 people (0.15%) who reviewed 10 or more manuscripts account for 1.6% of all reviews. Across the entire pool of reviewers, 11% reviewed for both journals. However, this measure underestimates the degree of overlap in the respective reviewer pools because the modal number of reviews by a reviewer is one. Out of the 12,129 reviewers who reviewed more than one manuscript at either journal, 4,638 (38%) reviewed articles at both journals. (The calculations in the paragraph are derived from the original, unfiltered data set.)

Manuscripts may go through any number of rounds of review, and there can be any number of reviewers in a given round. Typically, the same reviewers from the first round are re-invited in subsequent rounds and in practice most agree to participate again. However, the editor may, at their discretion, recruit new reviewers at any stage, e.g., to obtain complementary perspective on the manuscript or to replace a previous reviewer who is no longer available. Both journals have a policy of "rapid" peer review, in which reviewers are asked to return their reviews within a few weeks or a month of the initial invitation. (In practice, that request is more of an opening bid, and many reviewers ask for and are granted longer time to complete the review.) An editor may elect to terminate a peer review round before all promised reviews are received, e.g., if the first reviews that are returned indicate critical issues that are unlikely to be resolved through a revision. By journal policy, a "revise" decision after a round of peer review is based on the editor's assessment of the reviews and the manuscript together, and manuscripts that seem unlikely to succeed in peer review are supposed to be rejected sooner rather than later.

At both journals, accepted manuscripts are sent to the production process for publication. Accepted manuscripts at *Science* are typically published in print, which limits the total number of published articles per year. Accepted manuscripts at *Science Advances* are published online only, with no restriction on the maximum number of published articles in a year.

A brief history and commentary on the founding of *Science Advances*. *Science Advances* was created in 2014 and published its first issue in February 2015. It was created by AAAS to be an online-only general science journal with the same scope at *Science*, but edited by academic rather than professional editors and publishing only research articles (in contrast to the various essays, perspectives, and other "front matter" published by *Science*). *Science Advances* initially used a different manuscript management system than *Science*, but in 2017 began using the same system.

The creation of a new journal poses an interesting puzzle for a publisher: how should it best establish itself as a suitable home for high-quality manuscripts in the areas it covers? Authors are sensitive to journal reputation, and a new journal has no track record to give authors a sense of what type, range, or quality of manuscripts it publishes.

The stated goal of AAAS for *Science Advances* was to be a high-impact sister journal to *Science*, publishing a similarly broad range of topics and manuscripts of similarly high quality. In finding its place in the publishing ecosystem, *Science Advances* surely benefited from the existing strong reputation of *Science* and the marketing efforts of AAAS. That is, authors and communities who were already familiar with *Science* were probably more likely to consider sending manuscripts to *Science Advances* even when it was a new journal with an uncertain status and no track record. However, such an effect could not help the journal with authors and communities who were not already interested in *Science*, e.g., fields with their own established publication venue hierarchies like economics, computer science, and sociology.

Internally, the roughly 60 editors were told to be choosy, to select for manuscripts that were the highest quality interdisciplinary, multidisciplinary, or disciplinary contributions, even if it meant having a lower publication volume initially. Submission volume was relative modest at first, but grew steadily.

In 2018, *Science Advances* received its first journal impact factor of 11.7, which placed it among an elite group of high-impact general science journals like *Proc. Natl. Acad. Sci. USA* and *Nature Communications*. Over the subsequent year, the submission volume for *Science Advances* doubled. In 2019, the number of monthly submissions to *Science Advances* surpassed that of *Science*. By 2025, the editorial board had grown to more than 400 to manage the volume and breadth of submissions, which surpassed 28,000 per year in 2024.

Differences between *Science* and *Science Advances*. Because *Science* and *Science Advances* have such similar structures and scopes, the comparison of the two journals provides a unique opportunity to contrast the roles of professional and academic journal editors. The question of whether and how much it matters if a journal uses professional vs. academic editors has been a long-running debate in the academic and publishing community, albeit one that has lacked rigorous evidence *(41)*. The similarity of the results presented in this study for the two journals suggests that any real differences due to having academic or professional editors alone were not sufficiently large to overcome common factors that structured outcomes at both journals. However, we caution against over-interpreting differences, or the lack of differences, between the two journals as necessarily caused by the relative employment type of editors, because other editorial policies at the two journals could also drive observed or obscure genuine differences.

Understanding the extent to which editorial policies or the structure of editorial and peer review shape the outcomes of manuscript evaluations at publishing venues will require broader sources of evidence, and the results of experiments. Two examples of such experiments include initiatives at *eLife (42)* and at *F1000Research (43)*. For instance, in 2022, *eLife* adopted a model in which an editorial team makes the preliminary editorial review decision, and the manuscript is sent to review. However, rather than returning to make a final decision about publication, the reviewed manuscript is then guaranteed publication, and the reviews are published alongside the article. *F1000Research* takes this model further, and eliminates the initial editorial review stage altogether—manuscripts are published pre-review, and updated live with the reviews and subsequent editors from the authors.

Appendix S2: Data Specifications, Anonymization, and Augmentation

The anonymized data set contains a manuscript-level representation of the evaluation processes at *Science* and *Science Advances*, derived from a complete set of editorial records 2015–2020 provided by the American Association for the Advancement of Science (AAAS) through a Data Use Agreement. Only manuscripts that received a final decision by 1 January 2020 were included. The AAAS publishes several journals, but our analysis only included *Science* and *Science Advances*. We excluded commentaries, letters, and all submission types other than research articles. We drop 7769 reviews out of 88304 that lacked a written comment to the author and the 2.4% of submissions whose metadata indicated they were reviewed, yet didn't have any reviews attached. We additionally drop 13 submissions in which first-round reviews were listed as cross reviews, which are a type of supplementary reviewer-to-reviewer comment at *Science*.

In order to make this data set useful for the community, the attributes in each manuscript record were augmented and standardized. In order to protect the confidentiality of the peer review process at *Science* and *Science Advances*, personally identifiable and reidentifiable information was removed. The anonymized data set and anonymization scheme was then peer reviewed by an outside expert on anonymization procedures, recruited by AAAS for that purpose.

The procedures used to augment, standardize, and anonymize the data set are described in this section. Summary statistics of the resulting 110,303 manuscripts and their associated attributes are provided in Table S1.

The following is the full list of included variables for each manuscript record:

- IsAccepted
- Publication
- DeputyEditorGender
- AssociateEditorGender
- FirstAuthorGender
- CorrespondingAuthorPrestige
- CorrespondingAuthorGender
- CorrespondingAuthorRegion
- NumberOfAuthors
- BORERating1
- BORERating2
- BOREConfidence1
- BOREConfidence2
- BOREExists1
- BOREExists2
- BOREGender1
- BOREGender2
- ReviewSentiment1
- ReviewSentiment2
- ReviewSentimentTrajectory1
- ReviewSentimentTrajectory2
- ReviewLength1
- ReviewLength2
- Evaluation1
- Evaluation2
- Topic100
- Topic30
- Topic10
- ReviewExists1
- ReviewExists2
- ReviewerGender1
- ReviewerGender2
- ReviewerPrestige1
- ReviewerPrestige2

Future data sets on editorial and peer review.

None of these author or reviewer variables directly capture seniority or career stage. Future work creating anonymized peer review data sets should consider ways to add such a variable, e.g., years since Ph.D. or years since first publication, in order to account for the potentially unmodeled seniority effects discussed in the main text.

Author and reviewer demographic characteristics are limited to binary gender, which prevents analyses of other demographic variables or intersections among them. Extending these characteristics to include a richer range of demographic variables, most notably race, would facilitate further analyses of potential social biases in editorial or peer review, and their relation to other manuscript characteristics.

We used a relatively coarse definition of manuscript topic. Interviews with existing editors at *Science* and *Science Advances* suggest that topic alone is only one of several important dimensions relevant to editorial review, and that characteristics like novelty, broad vs. narrow utility, and relevance to extant literature are also important. Developing measures of these characteristics would facilitate investigations of how evaluation may operate, even in double-anonymous situations. Given the role of editors in shaping the distribution of topics in *Science* and *Science Advances*, a future data set focusing on editors could allow researchers to study the variance introduced through editor idiosyncrasies, as well as the role of editors in shaping how topic distributions change over the evaluation process.

Future data releases could summarize reviewer text using a panoply of methods from computational linguistics.

For instance, reviewer text could be reported with average word lengths, average sentence lengths, readability scores, and proportions of first vs. second vs. third-person pronouns. Re-identification risks proliferate with this type of data, because these features are similar to the ones used in stylometric analyses for identifying authors from text.

In the data provided, author suggestions for reviewers were not included, and nor was information about which reviewers were invited to review or the order in which they were invited, or any information about declared or identified conflicts of interest. Such data would be valuable to include to better understand how editors select which reviewers to recruit from the pool of potential reviewers.

Also not included in the data provided were the author responses to peer reviews. These documents are the key place where authors and reviewers debate the merits of the manuscript, and quantitative analyses of their rhetorical structure, along with analyses of the rhetorical structure of the reviews themselves, would shed considerable new light on how authors, reviewers, and editors negotiate criticism within peer review. Recent advances in natural language processing techniques represent a particularly promising way of making progress on these questions, if the data were available.

S2.1. Anonymization procedures

We dropped all identifying and confidential information, including article titles and abstracts; the names and other identifiers of all authors, reviewers, BoRE advisors, and editors; and the text of the peer reviews themselves. As a result, reviewers and authors cannot be linked across manuscripts within the data set, and the data set is at the manuscript-level. This design lowers the risk from cross-record reidentification attacks. A number of other attributes pose reidentifiability risks for a manuscript. To these, we apply a specific set of transformations in order to mitigate this risk.

The precise number of authors on a manuscript can be reidentifiable; hence, a coarse binning was applied to the raw team size values (1-5, 6-9, 10+) to produce the number of authors variable, and author characteristics were only retained for the first and corresponding authors. If a manuscript had only one author, the author information was duplicated for both first and corresponding author fields.

Records with multiple rounds of peer review or with many reviewers are particularly reidentifiable. Only 37.7% of reviewed submissions receive more than one round of peer review, as most are rejected after one round. To remove the potentially reidentifiable information of the number of rounds of review and the precise number of reviewers in the first round, in the de-identified data set, only information from the first round of reviews was included, for manuscripts that went to the peer review stage of evaluation. Within this included round, a maximum of two randomly selected first-round reviewers were included. Manuscripts that were rejected during editorial review have no associated reviews.

We note, the anonymized data does not link individuals across submissions (no person-unique identifiers), which mitigates cross-record reidentifiability risks.

Using modern natural language processing techniques, all manuscripts were clustered into broad topic areas based on their titles and abstracts. Topic labels for 10, 30, and 100 clusters are included. See Section S2.4 for more details.

Although the raw text of reviews is highly reidentifiable, the reviews themselves are a critical piece of the peer review stage and they are the only extant measure of manuscript quality available. We randomly selected up to two first-round reviews, and we summarized their text using statistical measures: their overall sentiment and their length were z-scored to further reduce their reidentifiability. See Section S2.5 for more details.

Reviewers do also submit ratings for "Overall evaluation" and a "Recommendation," each from a list of 5 options each, along with the text of their review (shared with authors), and comments to the editor (not shared with authors). A shallow audit of these ratings shows that reviewers often use them inconsistently, e.g., submitting a harshly negative review with an overall evaluation rating of "Above average" or submitting a positive review with an overall evaluation rating of "Too specialized." Parsing the review text itself was deemed necessary to properly contextualize the reviewer's use of these ratings. Hence, we include the review sentiment alongside the review evaluation. Reviewers can opt to submit a review evaluation of "No rating assigned". Finally, we assign a "Null" value to records that are not reviewed.

Similarly, for each manuscript, we include the numerical rating and confidence from up to two randomly selected BoRE advisors assigned to the manuscript. Manuscripts that were summarily rejected have no associated BoRE information.

Under a technical assessment of the anonymization scheme, it was determined that the most sensitive data that could be revealed for a given manuscript, were an individual record to be reidentified, would be the binned prestige scores and genders for the two randomly selected first-round reviewers, the gender and numerical ratings of (up to) two randomly selected BoRE advisors who evaluated the submission, and the genders of up to two editors.

S2.2. Authors, reviewers, BoRE, and editors

This section covers the following variables:

- DeputyEditorGender
- AssociateEditorGender
- FirstAuthorGender

Variable	What it applies to	Possible values
**Publication**	All submissions	*Science* (61.7%) / *Science Advances* (38.3%)
**Is accepted**	All submissions	TRUE (7.8%) / FALSE (92.2%)
**Number of authors**	All submissions	[1–5] 39.2%; [6–9] 29.6%; [10+] 31.2%
**Topic**	All submissions	1–N for $N = 10, 30, 100$
**Gender**	BoRE advisors	Man (76.6%) / Woman (23.4%)
	Editors	Man (65.8%) / Woman (34.2%)
	First authors	Man (66.6%) / Woman (33.4%)
	Corresponding authors	Man (78.5%) / Woman (21.5%)
	Reviewers	Man (77.2%) / Woman (22.8%)
**Prestige**	Corresponding authors	[1–2] 17.0%; [3–4] 17.5%; [5–6] 24.2%; [7–10] 28.2%; Null 13.0%
	Reviewers	[1–2] 28.0%; [3–4] 23.1%; [5–6] 21.5%; [7–10] 18.7%; Null 8.7%
**Region**	Corresponding authors	U.S. + Canada (31.3%), China (19.7%), Europe (24.5%), Other (17.2%), Null (7.4%)
**BoRE rating**	Most *Science* submissions	(lowest) 1–10 (highest)
**BoRE confidence**	Most *Science* submissions	(lowest) 1–5 (highest)
**Review evaluation**	Reviewed manuscripts	No rating assigned, Too specialized, Mediocre or poor, Average, Above average, Excellent and exciting, Null
**Review sentiment**	Reviewed manuscripts	Z-scores $[-3.5, 3.5+]$, coarsened and capped
**Review length**	Reviewed manuscripts	Z-scores $[-3.5, 3.5+]$, coarsened and capped
**Review trajectory**	Reviewed manuscripts	DF, DFU, DU, DUD, DUDF, F, FD, U, UD, UDF, UF, Null

TABLE S1. **Overview of data.** Rows show variables included in the anonymized data set. BoRE is the Board of Reviewing Editors. Topics are created through topic models of $N = 10$, 30, or 100 topics. Review lengths are the z-score of the numbers of words. Review sentiment trajectory values are concatenations of (D)own, (F)lat, and (U)p.

- CorrespondingAuthorPrestige
- CorrespondingAuthorGender
- CorrespondingAuthorRegion
- ReviewerGender1
- ReviewerGender2
- ReviewerPrestige1
- ReviewerPrestige2

For each included author (first and corresponding) and reviewer (maximum of two), we include their self-reported gender; when a self-report was missing, we included the label assigned by a name-based gender labeler *(44)*. The methodology we used assigned only binary (woman/man) labels to authors and reviewers, even as we recognize that gender is nonbinary. This approach represents a compromise due to the technical limitations of name-based gender methodologies *(45)* and is not intended to reinforce a gender binary. For editors and advisors who did not self-report genders, we manually assigned binary gender based on pronouns on institutional websites.

Among authors, 14.1% self-report gender. Out of the 14.1% authors who self-report gender, 99.8% self-report as either male or female. We found that 85.6% of authors were assigned the same label from the name-based labeler that they self-reported. Assuming that self-reporting is independent of the accuracy of the name-based labeler, then our overall accuracy is 88.5%, which assumes perfect accuracy for the self-reports, and 85.6% accuracy for the remainder. This accuracy could be raised by rejecting labels from the name-based labeler for which it was not confident (for example, the accuracy could be raised to 97.6% if we rejected 21.5% of predictions), but further stratification in the gender variable would introduce re-identification risk, which we sought to keep to a minimum.

For each included corresponding author and reviewer, we augmented their information by adding institutional prestige labels based on their self-reported affiliation or affiliation derived from an email addresses (see Section S2.3). For each corresponding author, we additionally include the affiliation's country region (see Table S1).

S2.3. Corresponding author and reviewer prestige

This section covers the following variables:

- CorrespondingAuthorPrestige
- ReviewerPrestige1
- ReviewerPrestige2

Our approach to add prestige variables to authors is motivated by the following two requirements.

First, we require an international measure of prestige, because only 31.3% of corresponding authors are from the U.S. and Canada (Table S1). This requirement precludes using standard prestige measures like those derived from U.S. faculty hiring networks *(46)*, and, currently, sufficient data on international faculty hiring is not available to populate a full global network.

Second, we require a measurement of prestige that extends beyond academic universities to encompass other institutions that frequently submit manuscripts to *Science* and *Science Advances*, such as hospitals, national research institutes, private research centers, and certain private companies. This requirement excludes the direct use of global university rankings like those produced by the U.S. News & World Report and the Times Higher Education.

To satisfy these joint requirements, we performed a hybrid approach that first employed an algorithmic linking to prior university rankings, followed by a coarsening to bins. We then solicited a diverse range of expert feedback on the rankings from an international set of scholars to annotate non-universities and adjust the prestige binning to reflect expert judgment.

We first algorithmically linked institutions listed in the manuscript records to the rankings of the US News & World Report (USNWR) Top 2000 Global Universities *(47)*. This approach required cleaning self-reported institutions using common natural language processing techniques, such as removing punctuation, parentheticals, and certain stopwords. This process was facilitated by a dictionary provided by AAAS with the original data that mapped 16,715 out of the 48,436 unique self-reported affiliations to a canonical institution name, which did not necessarily match the name provided by the USNWR rankings. To assist the linkage, we created a further set of manual synonyms for each university.

Viewed as a network where nodes are potential institution names, and edges are known connections between names (such as a canonical linkage provided by AAAS, a name that is a preprocessed version of another name, or a synonym that we generated ourselves), we de-duplicated the names by approximately inferring connected components of names on this network. In particular, we first checked for direct matches between self-reported institutions and the names provided by the USNWR rankings. Then, we expanded the set of canonical names for any given institution by associating with a given USNWR institution the set of aliases provided by AAAS and ourselves. Lastly, we matched any remaining self-reported institutions with aliases that were now linked to USNWR institutions. This approach matched 74.3% of author affiliations to the USNWR rankings. We sampled 54 unmatched institutions, and found that 15 (26%) should have been matched to the USNWR rankings, suggesting that our algorithmic approach has covered 92% of possible university-based matches.

To expand our rankings to include affiliations of submitting authors that were omitted from our algorithmic linkage, we focused on the most common institutions that remained unmatched.

By binning submissions by institution, we found that the top 1% of the unranked institutions, in terms of manuscripts submitted (292 institutions), accounted for 30.6% of the total unranked author-institution pairs. The top 5% of unranked institutions (1463 institutions) would only expand the total coverage to 50.9% of author-institution pairs. Thus labeling the top 292 institutions increased our coverage of prestige from 74.3% to 82% of all author-institution pairs. These 292 institutions included a mix of internationally renowned research institutes and institutions with virtually no international reputation, such as universities without a ranking in the USNWR Top 2000 Global Universities.

We labeled these remaining institutions, and checked the validity of the USNWR rankings, by sending a preliminary set of rankings to an international group of scholars. First, we binned the USNWR rankings into deciles, such that there were roughly an equal number of submissions from each decile. Thus the top of the hierarchy had fewer institutions than the bottom of the hierarchy, because the population of scholars who submit to *Science* and *Science Advances* are disproportionately based in the most prestigious institutions. Second, we assigned preliminary rankings to the missing institutions through the following set of rules.

- For foreign national labs, we assigned them the highest value of prestige in USNWR for universities in that country.
- When an institution had a clear university partner, we assigned that university's prestige to the institution.
- Well-known U.S. federal agencies were generally placed into the top two bins.
- Some private institutions with exceptional international reputations were placed into the top bin.

The remaining unrecognized institutions, such as universities that were omitted from the USNWR Top 2000 rankings, were placed in bottom bins.

These preliminary binned rankings were sent to 22 international experts, spanning a range of disciplines including but not limited to earth sciences, microbiology, cognitive science, applied mathematics, public health, data science, and physiology. Eleven of the experts were based in the U.S. or Canada, six were based in Europe, three were based in Asia, and the remaining two were

based in Australia and/or New Zealand. From this panel of experts, we received responses from 12, for a total of 195 proposed re-binning of institutions. We generally incorporated their suggestions, and when there were conflicts, we took the suggestion of the scholar who was geographically or topically closer to the disputed institution, using seniority as a tie-breaker.

We then coarsened the prestige labels by binning them: prestige bin 1–2 is the most prestigious group, followed by bin 3–4, then bin 5–6, and bin 7–10; institutions that were not assigned a prestige label were given a bin 'Null.' Finally, we note that the precise assignment of institutions to the final prestige bins presents a mild reidentification risk to the anonymized data set, and so we omit it.

S2.4. Article topic clusters

This section covers the following variables:

- Topic100
- Topic30
- Topic10

We assign each manuscript a topic at each of three levels of granularity: 100, 30, and 10 topics, and we use natural language processing techniques to learn these clusters directly from manuscript titles and abstracts. This approach ensures that the topics are both internally consistent with the data, and mitigates some risk of reidentification that may come from using an external topic taxonomy.

To produce these clusters, we first applied the publicly available SPECTER2 embedding model to submission titles and abstracts, yielding a vector of length 768 for each paper *(48)*. The SPECTER2 model was designed specifically to generate effective embeddings for downstream applications on scientific articles, e.g., two similar articles will produce vectors that are separated by a relatively small euclidean distance, while two dissimilar papers will produce vectors that are farther apart.

We then applied k-means clustering to each article's embedding vector in order to group nearby papers into the same topics. We ran this clustering for $k = 10$, $k = 30$, and $k = 100$, resulting in three sets of topic labels, in which every article is assigned to one of k clusters, with larger values of k representing more fine-grained distinctions between topics (and subtopics).

We note that the resulting topic labels do not necessarily adhere to a strict tree structure, meaning that, for example, two manuscripts assigned to the same topic when $k = 30$ may be assigned to distinct topics when $k = 10$. By providing multiple levels of granularity, researchers can choose a granularity appropriate for their research question.

We cannot release individual submission titles associated with each topic in the public data set due to data-privacy restrictions. To convey the topics represented by each Topic10 cluster, we extract a set of distinctive terms that characterize the research within each topic. To extract these terms, we standardized all submission titles using a synonym-normalization dictionary that merges plural forms and related multi-word expressions into canonical terms (e.g., *neuronal* to *neuron*). After normalization, the titles within each topic were concatenated into a single document and analyzed with a term frequency–inverse document frequency (TF–IDF) model configured with English stop-word removal and token filtering. The ten highest-scoring TF–IDF terms were selected as the most distinctive words for each topic. The resulting representative terms, shown in Table S13, provide an interpretable summary of the subject matter within each Topic10 cluster. We make available the full code used to generate topic labels, perform synonym normalization, compute TF–IDF representations, and extract the distinctive words reported here.

The inclusion of manuscript topic variables enables a variety of topic-based analyses. For instance, Figures 3 and S1 show the $k = 10$ topic-level manuscript success rates at each stage of editorial and peer review process at *Science* and *Science Advances* (respectively). These alluvial plots illustrate how the distribution of manuscripts over topics at the submitted stage differs substantially from the distribution of accepted manuscripts, with some topics shrinking in relative proportion (e.g., topics 8 and 3 in *Science Advances*) and others growing (e.g., topics 0 and 6 in *Science Advances*).

We find no consistent relationship between topic size and acceptance rates across the two publications (Fig. S2). Note this does not imply that editors do not curate the topic space of science; only that the extent of filtering a topic is uncorrelated with the number of submissions to that topic. Moreover, the topics are constructed in a data-driven fashion, and may not perfectly correspond with the types of topics that editors deem to be publishable or too specific. The editorial board size and composition itself also reflects the different sizes of topics.

S2.5. Review evaluation, sentiment, length, and trajectory

This section covers the following variables:

- Evaluation1
- Evaluation2
- ReviewSentiment1
- ReviewSentiment2
- ReviewSentimentTrajectory1
- ReviewSentimentTrajectory2
- ReviewLength1
- ReviewLength2

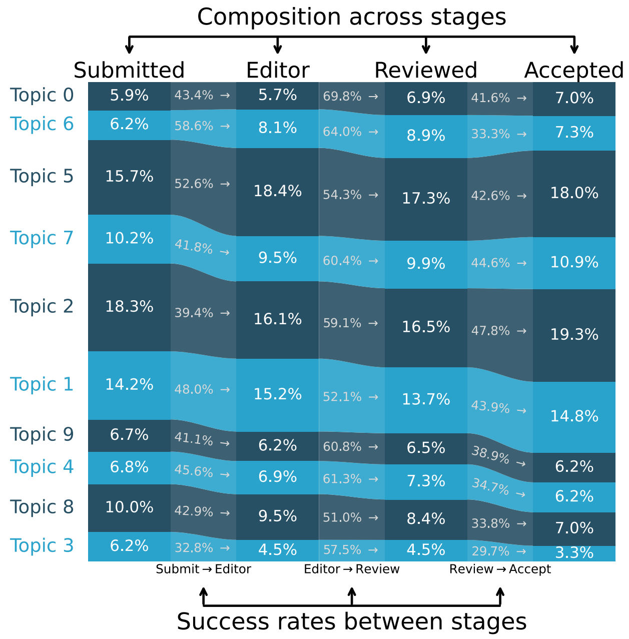

FIG. S1. **Topic representation across editorial and peer review stages at *Science Advances*.** Ten broad and de-identified topics are illustrated by their proportions among submitted, editor reviewed, peer reviewed, and accepted papers (white text) with success rates between stages calculated for reference (gray text). Topics are plotted in order from highest overall acceptance rate (topic 0) to lowest overall rate (topic 3). For distinctive terms that characterize each topic, see Table S13. *Science* shows similar results (Fig. 3).

For each review, we include the reviewer-chosen evaluation, and summarize its content using three measures: (i) overall review sentiment, (ii) review length, and (iii) the review's sentiment 'trajectory.'

The reviewer evaluation is a selection from one of six options: 'No rating assigned', 'Too specialized', 'Mediocre or poor', 'Average', 'Above average', or 'Excellent and exciting.' We leave them as categorical responses because there is no obvious way to order the evaluations into a single numerical variable.

The review sentiment variable measures the review's overall evaluation of the manuscript, while the sentiment trajectory quantifies the review's narrative structure from start to finish.

To extract overall review sentiments, we applied a Bidirectional Encoder Representations from Transformers (BERT) model that was fine-tuned for sentiment analysis on product reviews *(49)*. This model takes review text as input and returns the estimated probabilities that the review text is associated with a 1-star, 2-star, 3-star, 4-star, or 5-star review. We multiply these five estimated probabilities by their corresponding star-values, and sum the resulting products to construct a sentiment score that can range continuously from 1 (lowest possible sentiment) to 5 (highest possible sentiment). This model only considers the first 512 words of each review for evaluation. Even though this model was fine-tuned on product reviews, we found it performs well in measuring peer-review sentiment. We hand-annotated a random sample of 50 reviews for general sentiment on a scale from 1-10, considering the entire length of each review, and found that the sentiment analysis model's annotations strongly correlated ($r = 0.80$, $p < 0.001$). We then transform all review sentiments into z-scores, and then coarsen the z-scores by binning them at a resolution of 0.25 on a $[-3.5, 3.5+]$ range, with values outside that range mapped to the endpoints.

Review lengths were calculated in terms of the number of words in the review. We transform all review lengths into z-scores, and then coarsen the z-scores by binning them at a resolution of 0.25 on a $[-3.5, 3.5+]$ range, with values outside that range mapped to the endpoints.

A sentiment 'trajectory' variable measures the ebb and flow of review sentiment over the course of the review, which provides a coarse measure of the rhetorical structure of the review. For example, consider two hypothetical reviews that are both generally negative, and both ultimately recommend rejection. One review is negative throughout its entire length, and accordingly would have a flat sentiment trajectory. The other review begins on a positive note before before enumerating the paper's many flaws in a much more negative tone, resulting in a downward sentiment trajectory from start to finish. By quantifying such differences, this variable allows for simple analyses as to whether these two review narrative structures might have differing impacts on editor decisions. (An interesting direction for future work would develop more sophisticated "rhetorical decompositions" of reviews, e.g., Ref. *(50)*, to better understand how reviewer arguments influence editor decisions.)

To extract review sentiment trajectories, we used the same BERT sentiment analysis model as before on multiple 3-sentence segments of each review *(49)*. These segments were created by moving a 3-sentence window across the entire review, with each segment overlapping the next by 2 sentences. Thus, a 30-sentence review would yield 28 sequential sentiment measurements which form its sentiment trajectory.

To assign each review's sentiment trajectory to one of 11 narrative structures, we first normalized the trajectories by subtracting the sentiment associated with the first three sentences of the review from each point in the sentiment trajectory, so each trajectory starts with a sentiment score of 0. This step isolated the review's trajectory from it's overall sentiment, which is measured separately. Next, we normalized the lengths of the reviews by linearly interpolating 1000 evenly spaced points over the span of the trajectory. Accordingly, even if a trajectory is only 10 sentences long yielding only 8 sentiment estimates, the path of these 8 points are linearly interpolated to construct a 1000-point trajectory of the same shape. Finally, we performed k-means clustering with $k = 30$ on the normalized trajectories, yielding 30 narrative structure clusters or categories. We visualized

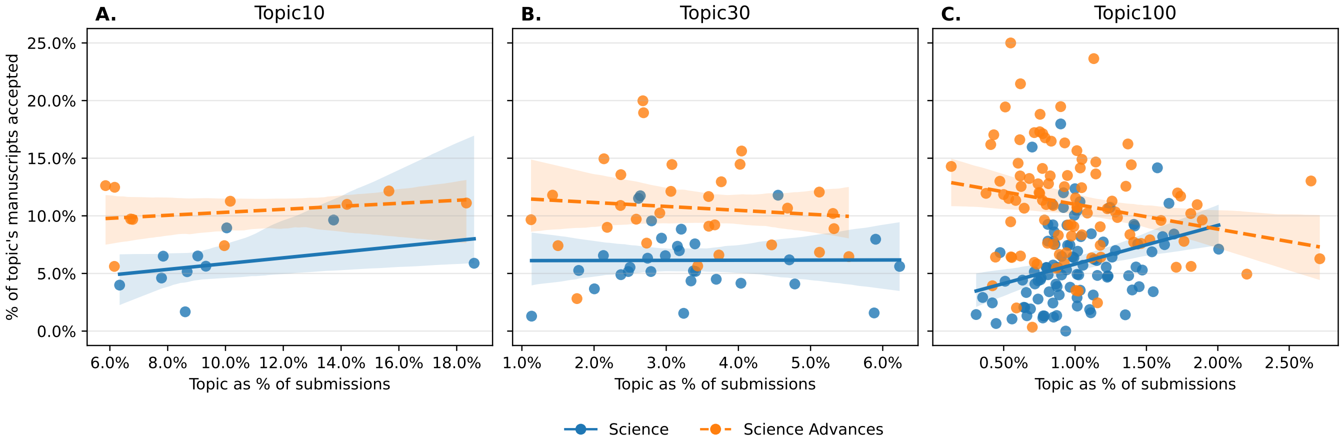

FIG. S2. **Acceptance rates by topic size.** Scatterplots with linear regression lines and 95% bootstrap CIs between the percentage of total manuscript submissions within a publication for a given topic on the x-axis and the acceptance rate for that topic on the y-axis, for *Science* (solid blue) and *Science Advances* (dashed orange). Panels are using (A) the topic10, (B) topic30, and (C) topic100 variables for the topic.

the mean trajectory for each of the clusters by plotting their centroids. We then further coarsened each cluster by assigning each of the 30 to one of 11 coarse-grained trajectories, defined by up (U), down (D) and flat (F) segments that we observed (Fig. S3).

To allow data users to make more informed decisions about whether and how to include these sentiment variables in their analyses, we make available code that can be used to apply the sentiment trajectory classification on user-defined input text.

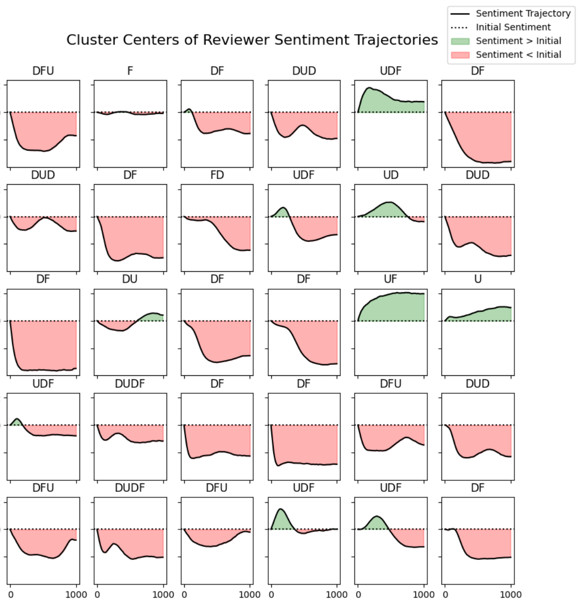

FIG. S3. Sentiment trajectories: K-means centroids for the 30 narrative structure clusters to which reviews are assigned. We coarsen these narrative structures by hand-labeling their various shapes into 11 groups, based on upward trends in sentiment (U), downward trends in sentiment (D) and flat spans of sentiment (F). See Section S2.5.

Appendix S3: Additional results on the correlates of success

For manuscripts in *Science Advances*, we repeat the multivariate logistic regression analysis for modeling the outcome of editorial review and peer review using available author and manuscript characteristics. Fig. S4 shows similar results to Fig. 2A in the main text for *Science*, in which institutional prestige for the corresponding author correlates the most with success in both the editorial and peer review stages, followed by region and team size, with more modest correlations for gender.

However, we observe a few notable differences in the *Science Advances* results relative to *Science*. The team-size effect at *Science Advances* is slightly smaller than at *Science*, at both stages of review. In addition, the prestige effect is slightly smaller in editorial review, but larger in peer review. And, we find a larger disadvantage to being sent for review when the Deputy Editor is a woman.

Table S8 shows the results of logistic regression models of the likelihood of a manuscript being sent to review at *Science* and at *Science Advances*, as a function of manuscript attributes and author characteristics. However, the likelihood of being sent for review at *Science* correlates strongly with the BoRE advisor ratings (Fig. 1C). Table S9 shows the results of the logistic regression model for this outcome of editorial review with controls for BoRE advisor ratings.

For manuscripts that pass through editorial review and

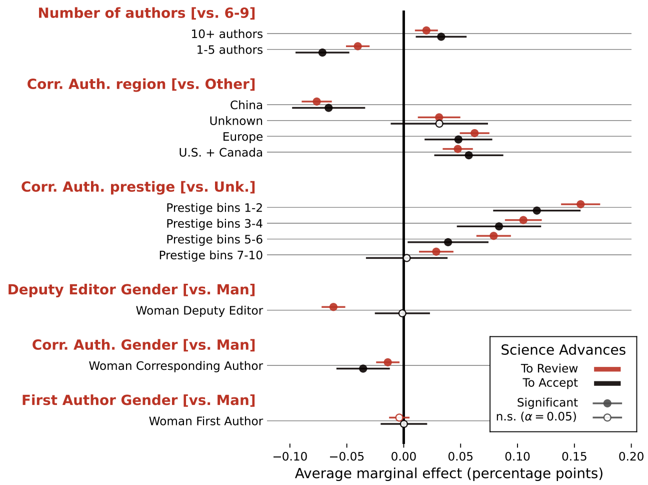

FIG. S4. **Manuscript characteristics and success in two stages of review.** Average marginal effects for each non-topic manuscript covariate at *Science Advances*, at two stages: the editorial decision to send a manuscript to peer review (red) and the final editorial decision to accept a manuscript for publication (black). Reference categories for each covariate are shown in brackets in the covariate label; statistical significance is indicated with a solid marker (z-test, $\alpha = 0.05$), and error bars display 95% CIs.

receive peer reviews, Table S10 shows the results of linear regression models of the review sentiment at *Science* and *Science Advances*. Fig. S5 shows the correlates of review sentiment for *Science* and *Science Advances*, using OLS regression with cluster-robust standard errors. These results illustrate the substantial variation in review sentiment by topic, and otherwise largely align with the results shown in Figs. 4 and S4.

Table S11 shows the results of logistic regression models of the probability of being accepted, at *Science* and at *Science Advances*. However, this probability strongly correlates with review evaluation and sentiment (Fig. 1D). Table S12 shows the results of the logistic regression models for this outcome of peer review with controls for review sentiment and evaluations.

To establish the percentage of the acceptance rate difference in corresponding author gender and team size explained by topic, we first compute the overall difference in acceptance rates by those covariates without any controls. Then, we compute the counterfactual difference in acceptance rates if the small teams with 1–5 authors (or women corresponding author teams) had submitted to the same topic distribution as the large teams with 10+ authors (or men corresponding author teams), assuming the acceptance rates within topics by gender would remain constant. At *Science*, the acceptance rate for 10+ authors was 9.94%, and for 1–5 authors was 3.30%. If the topic distribution for papers with 1–5 authors had been the same as that for those with 10+ authors, then the counterfactual acceptance rate would have been 3.90%, explaining 9.0% of the difference. At *Science Advances*,

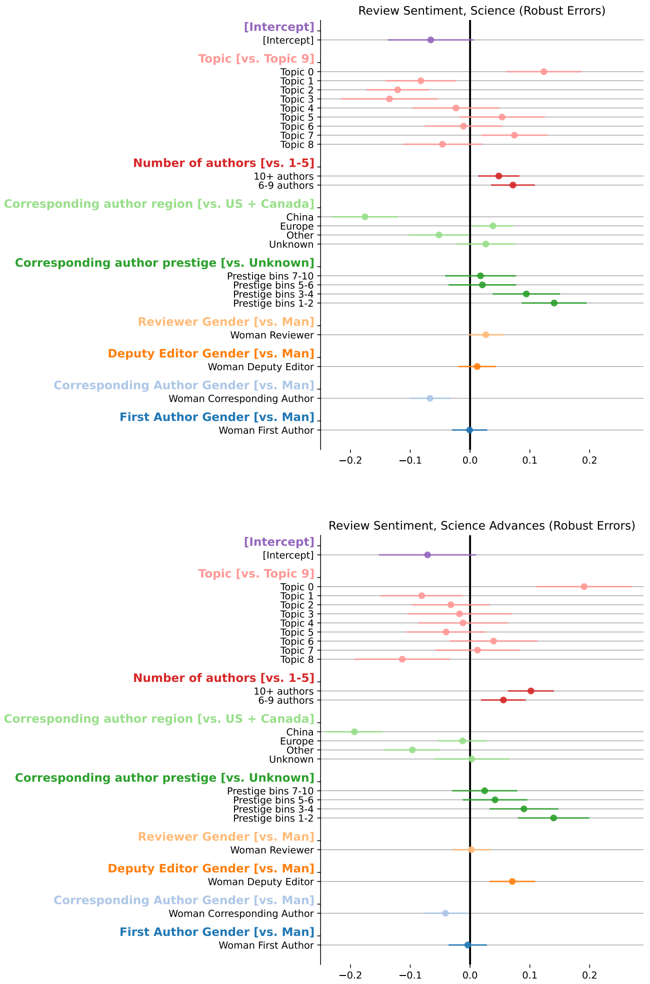

FIG. S5. **Review sentiment regression with cluster-robust standard errors.** OLS regression on review sentiment at *Science* and *Science Advances* where each record is a single review-manuscript pair, and standard errors are cluster-robust for each manuscript. Topic 4 is selected as the intercept instead of topic 0 because sentiments in topic 0 tended to be much higher than every other topic. Error bars display 95% CIs.

small teams had an acceptance rate of 8.21% and large teams 12.52%; controlling for topic led to a counterfactual small team acceptance rate of 8.13%. Reweighting by topic does not explain any of the team-size gap at *Science Advances*; if anything, small teams submit to slightly more favorable topics than large teams.

For women corresponding author papers at *Science*, the acceptance rate is 5.26% compared to men, with 6.38%; counterfactually, if women submitted with the same topic distribution as men, the acceptance rate would be 5.41%, explaining a total of 13.1% of the difference. At *Science Advances*, the acceptance rate is 9.54% for women corresponding authors, and 10.84% for men; after controlling for topic, it is 9.60% for women, accounting for 4.3% of the difference.

Appendix S4: Mediation analysis

To untangle the relative contribution of the editor in creating these correlations, we performed a mediation analysis, decomposing the Total Effect (TE) of each attribute on either desk rejection or final acceptance into a mediated component that is driven by expert evaluation (the Average Causal Mediation Effect, or ACME) and a component for the editor (the Average Direct Effect, or ADE).

We first performed this analysis on the set of all submissions to *Science*, using the rating assigned by the BoRE advisors as the mediator, and whether the manuscript was sent to review or desk rejected as the outcome (Table S2). Next, we performed the mediation analysis on the set of reviewed papers to both journals, using the sentiment scores of the first round peer reviews as the mediator, and whether the paper was accepted or rejected as the outcome (Table S3). We selected sentiment rather than reviewer evaluation as the mediating variable because it is continuous, whereas the use of evaluation, a nominal variable with six categories, would

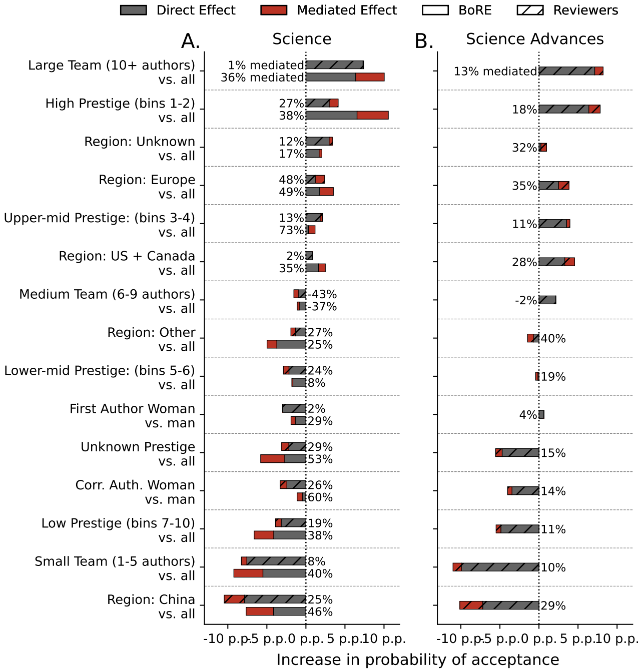

FIG. S6. **Expert mediation of editor decisions.** Decomposition of the increase in probability of acceptance, controlling for available covariates, into the portion attributable directly to the editor (gray) vs. mediated by the expert (red) for selected covariates in the (A) editorial and peer review stages at *Science*, and (B) the peer review stage at *Science Advances*. At *Science*, the BoRE ratings mediate the editor's decision in editorial review (solid bars), and the review sentiments mediate the editor's final decision in peer review (striped bars). The percentage of the total effect that is mediated by the expert is written for each stage and covariate.

have been difficult without restricting the analysis to certain responses. Selected results from these analyses are shown in Fig. 5 in the main text; a more comprehensive list of results are shown in Fig. S6. Below we provide addition discussion of the results and their interpretation. We note that Fig. S6 also shows comparable analysis for *Science Advances*, but only for the second stage.

The mediation analysis at the desk rejection stage reveals that the BoRE drives a significant share of the correlation at *Science* between peer review success and author prestige (37.9% mediated), author region (41.7% – 45.8% mediated), and team size (36.4% - 40.3%). Nevertheless, editors tend to drive the majority of the correlation associated with these attributes.

One exception to this pattern is that the majority of the TE associated with the corresponding author gender is mediated by the BoRE (61.2%).

The second mediation analysis reveals similar patterns at the stage of the editors' final decisions. At this stage, editors also drive the majority of the observed correlations, but to a higher relative degree, where the review sentiment never mediates more than 33.1% of the TE (such as the effect of a corresponding author belonging to an institution in the US, Canada, and Europe), and is often much smaller, where the ADE constitutes almost the entire TE for certain covariates (such as the role of large team size at *Science*).

One explanation for this pattern is that peer review occurs on the sample of data already selected by the editors during editorial review. If editors select manuscripts based on either observable author demographics D or manuscript quality Q, then the selection itself will condition on a confounder, and complicate efforts to untangle disparities downstream (Fig. S7B). Thus, attenuation of the effect of any demographics on manuscript outcome between the editorial and peer review stages may occur due to statistical reasons, even if editors and reviews acted with the same preferences.

However, the presence of collider bias does not foreclose the possibility of alternative mechanisms explaining differences between editorial and peer review outcomes. One such alternative explanation is that greater attention toward the content of the manuscript could reduce the correlation between author and team characteristics and outcomes. Author and team characteristics influence manuscript evaluations in two ways: (a) they can directly impact the evaluations due to their signaling value, which includes implicit gender bias and reputational effects; or (b) they correlate with the text of the manuscript, reflecting deeper differences in the scientific content or presentation. If (a) is a larger effect than (b), then increasing the attention toward a manuscript should decrease the correlation between author and team characteristics and outcomes. This assumption aligns with past research which showed that author characteristics influenced peer review outcomes more for brief reports than for full articles, suggesting that more research content reduces the reliance on author characteristics *(26)*.

Treatment	TE	ADE	ACME	Prop. Mediated
Corresponding author woman	-0.011*<br>(-0.02, -0.002)	-0.004<br>(-0.013, 0.004)	-0.007*<br>(-0.01, -0.003)	60.5%*<br>(26.9%, 235.4%)
Prestige (top vs. all)	0.106*<br>(0.096, 0.116)	0.066*<br>(0.056, 0.074)	0.04*<br>(0.036, 0.044)	37.8%*<br>(34.1%, 41.8%)
China vs. all	-0.077*<br>(-0.088, -0.067)	-0.042*<br>(-0.053, -0.031)	-0.035*<br>(-0.039, -0.032)	45.3%*<br>(38.7%, 53.9%)
US, Canada, and Europe vs. all	0.057*<br>(0.049, 0.065)	0.033*<br>(0.026, 0.041)	0.024*<br>(0.021, 0.027)	41.8%*<br>(35.9%, 48.8%)
Small team (1-5 authors vs. all)	-0.092*<br>(-0.1, -0.084)	-0.055*<br>(-0.062, -0.047)	-0.037*<br>(-0.04, -0.034)	40.3%*<br>(36.8%, 44.6%)
Big team (10+ authors vs. all)	0.1*<br>(0.092, 0.109)	0.064*<br>(0.056, 0.072)	0.037*<br>(0.034, 0.04)	36.5%*<br>(33.5%, 40%)
Topic (Topic 3 vs. all)	-0.039*<br>(-0.055, -0.022)	-0.006<br>(-0.021, 0.01)	-0.033*<br>(-0.04, -0.028)	85%*<br>(61%, 144.8%)

TABLE S2. **Mediation analyses for BoRE ratings.** Analysis of the probability of being sent to review mediated by BoRE ratings, showing the Total Effect (TE), Average Direct Effect (ADE), Average Causal Mediation Effect (ACME), and the proportion mediated. Results are specific to *Science* because *Science Advances* does not use a BoRE. The proportion mediated indicates the proportion of the total effect that is transmitted through the BoRE ratings. Each of the treatments are the indicated category contrasted with all of the remaining data. For example, the prestige analysis is the corresponding author belonging to the top bracket of prestige (bins 1-2) contrasted with all of the remaining authors. Parentheses show 95% confidence intervals and asterisks indicate that the 95% CIs do not overlap with zero.

Treatment	Journal	TE	ADE	ACME	Prop. Mediated
Corresponding author woman	Science	-0.033*<br>(-0.054, -0.013)	-0.024*<br>(-0.045, -0.005)	-0.008*<br>(-0.013, -0.004)	25.5%*<br>(11.4%, 65.4%)
	Science Advances	-0.04*<br>(-0.062, -0.019)	-0.034*<br>(-0.056, -0.014)	-0.006<br>(-0.011, 0)	14.1%<br>(-0.4%, 35.1%)
Prestige (top vs. all)	Science	0.041*<br>(0.021, 0.06)	0.03*<br>(0.011, 0.048)	0.011*<br>(0.007, 0.016)	27.9%*<br>(16.6%, 51.4%)
	Science Advances	0.078*<br>(0.053, 0.104)	0.064*<br>(0.039, 0.089)	0.014*<br>(0.008, 0.021)	18.5%*<br>(10.5%, 29.2%)
China vs. all	Science	-0.104*<br>(-0.131, -0.074)	-0.078*<br>(-0.105, -0.049)	-0.026*<br>(-0.032, -0.019)	25%*<br>(17.1%, 36.7%)
	Science Advances	-0.101*<br>(-0.121, -0.078)	-0.071*<br>(-0.091, -0.049)	-0.03*<br>(-0.036, -0.023)	29.5%*<br>(22.1%, 38.8%)
US, Canada, and Europe vs. all	Science	0.034*<br>(0.013, 0.053)	0.022*<br>(0.002, 0.041)	0.011*<br>(0.007, 0.016)	34%*<br>(19.1%, 83.2%)
	Science Advances	0.07*<br>(0.053, 0.088)	0.048*<br>(0.032, 0.067)	0.022*<br>(0.017, 0.027)	30.9%*<br>(22.5%, 41.8%)
Small team (1-5 authors vs. all)	Science	-0.083*<br>(-0.101, -0.066)	-0.076*<br>(-0.093, -0.059)	-0.007*<br>(-0.011, -0.003)	8.6%*<br>(3.8%, 13.9%)
	Science Advances	-0.11*<br>(-0.128, -0.092)	-0.099*<br>(-0.116, -0.081)	-0.011*<br>(-0.016, -0.006)	10.1%*<br>(5.4%, 14.7%)
Big team (10+ authors vs. all)	Science	0.075*<br>(0.058, 0.092)	0.074*<br>(0.058, 0.089)	0.001<br>(-0.003, 0.005)	1%<br>(-4.4%, 6.3%)
	Science Advances	0.082*<br>(0.064, 0.1)	0.072*<br>(0.054, 0.089)	0.011*<br>(0.006, 0.015)	13.3%*<br>(7.6%, 20%)
Topic (Topic 3 vs. all)	Science	-0.123*<br>(-0.159, -0.087)	-0.107*<br>(-0.144, -0.07)	-0.016*<br>(-0.025, -0.007)	13%*<br>(5.6%, 23.1%)
	Science Advances	-0.1*<br>(-0.141, -0.061)	-0.097*<br>(-0.136, -0.058)	-0.003<br>(-0.015, 0.008)	3.3%<br>(-9.9%, 15%)

TABLE S3. **Mediation analyses for review sentiment.** Analysis of how acceptance among reviewed papers is mediated by review sentiment, showing the Total Effect (TE), Average Direct Effect (ADE), Average Causal Mediation Effect (ACME), and the proportion mediated. The proportion mediated indicates the proportion of the total effect that is transmitted through the review sentiment. Each of the treatments are the indicated category contrasted with all of the remaining data. For example, the prestige analysis is the corresponding author belonging to the top bracket of prestige (bins 1-2) contrasted with all of the remaining authors. Parentheses show 95% confidence intervals and asterisks indicate that the 95% CIs do not overlap with zero.

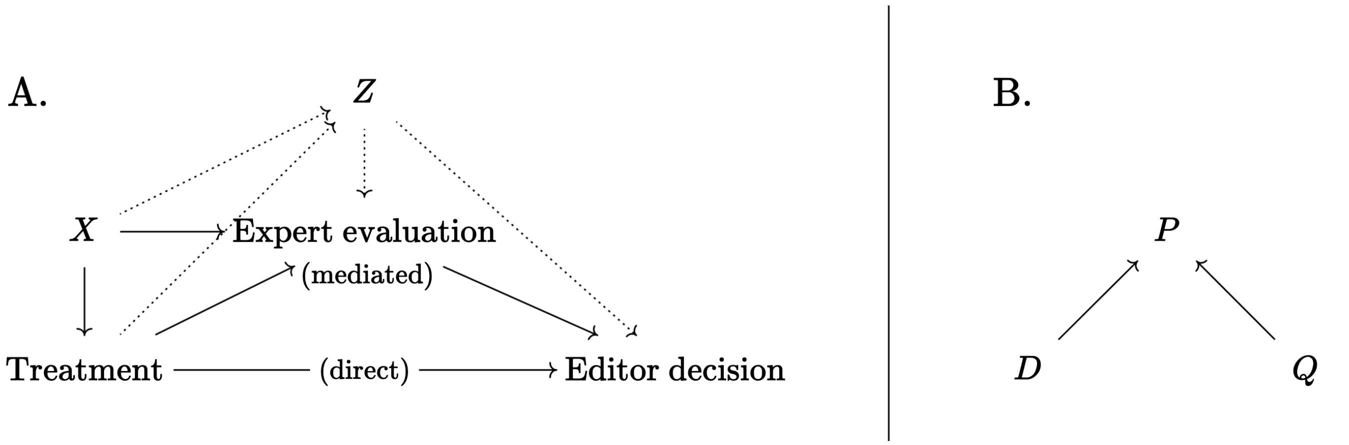

FIG. S7. **Causal diagrams of the mediation analysis.** (A) For every given treatment variable, we analyze the degree to which the total effect of the treatment on the editor's decision (whether the manuscript is sent to review or accepted) is mediated by expert evaluation (BoRE ratings or review sentiment respectively), as opposed to directly affecting the decision through the editor's decision-making independent of the expert evaluation. We assume that we have controlled for all relevant pre-treatment confounders X, and assume the non-existence of post-treatment confounders Z. (B) A stylized depiction of the collider that could complicate analyses of disparities at the peer review stage. If manuscripts are selected during editorial review due to either observable demographic information D or quality of manuscript Q, then simply passing editorial screening P is conditioning on a collider.

The attentional hypothesis is consistent with the observation that the experts (the BoRE advisors and peer reviewers) often read submissions in greater detail than the editors at their respective stages, and also have closer subject matter expertise to the manuscript. For example, an editor may be more sensitive than a reviewer to the reputational cost to the journal of rejecting a paper from a larger or highly prestigious team. Moreover, the fact that peer reviewers are responsible for evaluating manuscripts in substantially greater depth than even the BoRE advisors is consistent with the result that reviewers are proportionally less responsible for the introduction of correlations between author and team characteristics and final outcomes than the BoRE advisors during the editorial review stage.

Another possible mechanism driving the low proportion of mediation at the final acceptance stage is that editors at *Science* and *Science Advances* select manuscripts that they consider to be interesting for a general audience, while the reviewers may be relatively more focused on the technical correctness of the manuscript. For example, larger teams work on different types of science than smaller teams *(31)*, but the difference in these types of science may be more salient to editors than reviewers, leading to editors driving a larger proportion of the effect of team size on final acceptance.

These data do not support an analysis to identify which (if any) of these proposed mechanisms contribute to the observed pattern, in which the editor-driven ADE makes up high proportions of the TE for prestige, country, gender, and team size. However, these results can shed light on where potential interventions may be more or less effective. For example, transitioning to double-anonymous peer review, in which the expert evaluators do not directly observe a submission's author information, has the potential to decrease any effects due to viewing the author and team characteristics, but not the effects that are driven by the manuscript content. Thus our results can be interpreted as a possible upper bound for the effectiveness of blinding BoRE advisors and peer reviewers, because we quantify the extent to which editors could still introduce correlations between author characteristics and editorial outcomes even if the authors were anonymous to the experts. The finding that the BoRE mediates 37.9% – 61.2% of the total effects associated with team attributes, while the sentiment of the reviews only mediates up to 33.1% of the total effects, suggests that blinding the BoRE could result in a particularly large change to the observed total effects relative to blinding the peer reviewers.

Our mediation analysis uses the general causal mediation approach of Imai, Keele, and Tingley (2010) *(51)* implemented in the R `mediation` package *(52)*. Each record is a single rating-manuscript or review-manuscript pair, and standard errors are clustered by manuscript. Estimates are obtained using the default quasi-Bayesian Monte Carlo method based on normal approximation using 1000 Monte Carlo simulations. The outcome model that predicts final outcome using all of the covariates and the mediator employs the probit link to make it compatible with the `mediation` package, which only performs sensitivity analyses for binary outcomes using a probit link. This approach assumes *sequential ignorability*, meaning that the treatment is assumed to be ignorable given pre-treatment covariates, and that the mediator is ignorable given both the observed value of the treatment and the pre-treatment covariates, which implies that all of the relevant pre-treatment covariates are measured and that there are no post-treatment confounders. See Fig. S7A.

As a result of how the mediation analysis views the various authorship attributes as the treatments, any pre-treatment confounder must drive both the expert evaluation as well as the authorship attribute. Due to the long timescales on which most of the author attributes are fixed (such as the geographical region of the corresponding author), we believe that the only author attributes that could be plausibly influenced by an unobserved pre-treatment confounder in our analysis are the team sizes, where possibly the choice of research question could drive both research team size becoming larger or smaller as well as peer review outcomes in a way that is not captured by our topic variables. To account for this possible unmeasured confounder, we performed a sensitivity analysis using the `medsens` function from the `mediation` package, using the default choice of 1,000 Monte Carlo samples for the computation. The sensitivity parameter of interest is the correlation ρ between the residuals of the mediator regression and the outcome regression, which we vary between 0 and 1 at intervals of 0.1, and perform linear interpolation to estimate the value of ρ where the confidence interval of the ACME first contains 0. We find that the ACME remains outside of 0 for any of the authorship covariates across both journals as long as $\rho > 0.29$.

The mediation analyses rely on two models. The first is a linear regression that predicts reviewer sentiment, controlling for the region, gender, and prestige of the corresponding author; the gender of the first author; the number of authors; and the manuscript topic binned at the topic-10 level. The second is a logistic regression that predicts the final acceptance, controlling for the same covariates, as well as the reviewer sentiment. The mediation is performed using 1000 Monte Carlo draws using a quasi-Bayesian approximation, with standard errors clustered at the manuscript level.

The outcome model for the BoRE mediation analysis performs a probit regression on whether the manuscript was sent for review using the manuscript topic (at the level of granularity with ten topics, `Topic10`); the region, gender, and prestige of the corresponding author; the gender of the first author; the number of authors; and the BoRE rating. The mediation model for the BoRE analysis uses a linear regression to predict the BoRE rating using topic; corresponding author region, gender, and prestige; first author gender; and the number of authors. The outcome and mediation models for the final accep-

Journal	Evaluation Steps with Gender Disparities Counterfactually Removed	Observed Num. Articles with Women CAs	Counterfactual Num. Articles with Women CAs (95% C.I.)	Change Factor Under Intervention (95% C.I.)	
Science	None	750	752 (702, 800)	1.00 (0.94, 1.07)	
Science	Reviewed	750	788 (739, 839)	1.05 (0.99, 1.12)	
Science	Review Sentiment	750	765 (716, 816)	1.02 (0.95, 1.09)	
Science	Accepted	750	789 (740, 838)	1.05 (0.99, 1.12)	
Science	Review Sentiment, Accepted	750	803 (755, 856)	1.07 (1.01, 1.14)	*
Science	Reviewed, Accepted	750	827 (775, 877)	1.10 (1.03, 1.17)	*
Science	Reviewed, Review Sentiment, Accepted	750	842 (791, 889)	1.12 (1.05, 1.19)	*
Science Adv.	None	898	902 (848, 953)	1.00 (0.94, 1.06)	
Science Adv.	Reviewed	898	940 (889, 993)	1.05 (0.99, 1.11)	
Science Adv.	Review Sentiment	898	912 (857, 965)	1.02 (0.95, 1.07)	
Science Adv.	Accepted	898	952 (898, 1002)	1.06 (1.00, 1.12)	
Science Adv.	Review Sentiment, Accepted	898	965 (914, 1017)	1.07 (1.02, 1.13)	*
Science Adv.	Reviewed, Accepted	898	995 (942, 1045)	1.11 (1.05, 1.16)	*
Science Adv.	Reviewed, Review Sentiment, Accepted	898	1005 (946, 1064)	1.12 (1.05, 1.18)	*

TABLE S4. **Counterfactual analysis of gender.** CA = Corresponding Author. For each journal and selected steps of evaluation, we simulate predicted numbers of accepted papers after counterfactually removing gender disparities in that subset of steps. Shown are the observed number of women CAs, the counterfactual number of women CAs (with 95% CIs from Monte Carlo simulation), and the change factor under intervention with 95% Monte Carlo CIs. Change factor is the ratio of counterfactual women CAs to observed women CAs. Asterisks in the right-hand column indicate statistical significance, meaning that the 95% confidence interval of the change factor excludes 1.0.

tance analysis are identical, except that the BoRE rating is replaced with the review sentiment, and the data is restricted to the subset of manuscripts that were sent to review.

Appendix S5: Counterfactual model of no gender disparity in evaluations

To estimate the effect sizes of disparities by corresponding author gender at *Science* and *Science Advances*, we conducted several analyses that modeled counterfactually gender neutral outcomes, compounded over the evaluation process (Table S4).

First, to estimate how article attributes correlate with peer review outcomes, we ran regression models for desk rejections (editorial review), review sentiment (peer review), and manuscript acceptance (peer review) separately for *Science* and *Science Advances*. The decision to send an article to review (editorial review) and the decision to ultimately accept an article (peer review) each have binary outcomes, so we ran logistic regression at these steps. Peer review sentiment scores are continuous, so we ran linear regression for this outcome variable. For each regression, we included first author gender, corresponding author gender, deputy editor gender, corresponding author prestige, corresponding author region, number of authors, and topic as categorical predictors. For the regression analyses that correspond with the editor's final decision to accept or reject each paper, we additionally included the review sentiment from one of the reviews as a continuous predictor.

Then, we used the parameters inferred by these models to compute the estimated outcomes for each submitted manuscript at each step of the evaluation process, regardless of whether the manuscripts actually made it past editorial review. To estimate the probability of desk rejection in editorial review, we applied the corresponding regression equation, which was empirically fit based on observed desk rejection outcomes, to all submissions. To estimate review sentiment scores for each manuscript, we probabilistically draw from the prediction interval for each record based on the OLS regression fit to peer review sentiment outcomes. Here, we discard the empirical peer review sentiment scores, for the cases in which a given manuscript actually did go to peer review. Finally, we estimate the probability of acceptance (conditioned on being reviewed), based on observed covariates and the model-based review sentiment scores drawn in the prior step.

To estimate gender-neutral outcomes at a given step of the evaluation process, we modify records with women corresponding authors to counterfactually have man corresponding authors. This counterfactual switch has the effect of increasing the estimated probability of success (or increasing review sentiment scores) for women authored publications because the coefficient associated with women corresponding authors was negative across stages for both *Science* and *Science Advances* (Fig. 4).

We use this process of inferring success probabilities and of drawing review sentiment scores to run 1000 Monte Carlo simulations for each scenario and each journal, as outlined in Table S4. We first compute the estimated probabilities that each manuscript goes to re-

view, considering whether women corresponding author's records should be counterfactually altered at this step, based on the given scenario. Then, to prevent the journals from counterfactually publishing more papers, we scale the estimated probabilities, such that the sum of probabilities is equal to the observed number of reviewed manuscripts. Then, desk rejections are simulated based on Bernoulli-distributed weighted coin-flips. Review sentiments are then drawn for articles that were not desk rejected during editorial review, which are used as one of the covariates for the model fitted to final acceptance decisions. Before being used to weight another set of coin-flips to determine simulated acceptances, the final acceptance decision probabilities are scaled to sum to the total number of observed acceptances. Scaling the probabilities causes the the simulated number of acceptances to match the observed number of acceptances in expectation, across stochastic trials.

At the end of each trial, we record the simulated number of articles with woman corresponding authors, which we use to calculate the mean counterfactual number of manuscripts with women corresponding authors, along with confidence intervals spanning the middle 95% of Monte Carlo outcomes (Table S4).

Appendix S6: Testing drivers of women author disadvantage

To measure potential interactions between reviewer gender and corresponding author gender at *Science* and *Science Advances* in the resulting review sentiments, we estimate differences in review sentiment, grouped by six pairings of corresponding author gender and reviewer by gender (Fig. 6).

For example, we compare the sentiment scores for articles authored by a man and reviewed by a man ("MM") to articles authored by a man but reviewed by a women ("MW"). This design allows us to assess relative differences in review sentiments, and specify multiple hypotheses for how reviewer gender may interact with author gender.

We use propensity score weighting to control for covariate differences between comparison groups. We check the robustness of our results by separately running the analysis using both regular logistic regression *(53)* (as presented in Fig. 6 and Table S5) and gradient boosted logistic regression (see Table S6) to estimate propensity scores *(54)*. Through these models, we account for first author gender, deputy editor gender, corresponding author prestige, corresponding author region, number of authors, review length, topic (one-hot encoded into 10 categorical topic variables), reviewer gender, and reviewer prestige.

We then use average treatment effect (ATE) weighting in a weighted linear regression, with review sentiment scores as the outcome variable. For this regression, we once again control for the same set of covaraites that were used to estimate propensity scores, to be doubly-robust *(55)*. This regression estimates the ATE, which captures the direction and magnitude of the average difference in sentiment score between groups (net of the controls).

We use this approach to estimate the average difference in sentiment score and the corresponding statistical significance for each of six unique author-reviewer gender pairing: MM vs. MW, MM vs. WM, MM vs. WW, MW vs. WM, MW vs. WW, and WM vs. WW (Fig. 6).

We present results that correct for multiple hypothesis testing using the Bonferroni correction by dividing the significance threshold (0.05) by the number of statistical hypotheses conducted for each publication–6 in this case–yielding a corrected threshold of 0.0083. Under this correction, the results for *Science* were unchanged, and the uncorrected results for *Science Advances* changed to become entirely non-significant. In Tables S5 and S6, we additionally present results under the the Benjamini-Hochberg correction *(56)*, which is more permissive of significant results.

While the uncorrected and Benjamini-Hochberg corrected results based on propensity scores estimated using gradient boosted logistic regression (Table S6) match the results based on regular logistic regression (Table S5, two of the estimated pair-wise differences based on gradient boosted scores for *Science* become insignificant under the Bonferroni correction: MM vs. WW, and MW vs. WW (Table S6).

The results presented in Fig. 6 for *Science* show that teams with men corresponding authors receive more positive reviews than teams with woman corresponding authors, regardless of the gender of the reviewer. It is worth nothing that the de-identified data set cannot untangle whether this is the result of a boost for men corresponding authors or a penalty for women corresponding authors, and it is consistent with an unmodeled seniority effect for men (see main text for discussion).

We make available the code we used to run these analyses.

Appendix S7: Chinese name analyses

To assess the correlation between having Chinese names and manuscript outcomes, we conducted a pair of analyses.

Using the unanonymized editorial data, we first collected the 150 statistically most common surnames from authors who submitted with China-based affiliations, which accounted for 96% of the authors from China-based institutions in the database. Then, we labeled individuals affiliated with institutions located in the U.S. and Canada as having either a Chinese or a non-Chinese surname, based on whether it belonged to one of the 150 names extracted in the first step, labeling 107,147 (79%) as having a non-Chinese surname, and 29,272 as having a Chinese surname (21%). By comparison, only 5.4% of

Pair 1	Pair 2	Pub.	Not Corrected	Bonferroni	Benjamini-Hochberg	Coef.	p	Pub.	Not Corrected	Bonferroni	Benjamini-Hochberg	Coef.	p
MM	MW	Science	n.s.	n.s.	n.s.	-0.012	0.483	Science Advances	n.s.	n.s.	n.s.	-0.006	0.774
MM	WM	Science	>	>	>	0.075	0.000	Science Advances	>	n.s.	>	0.048	0.026
MM	WW	Science	>	>	>	0.120	0.002	Science Advances	n.s.	n.s.	n.s.	0.062	0.107
MW	WM	Science	>	>	>	0.099	0.000	Science Advances	n.s.	n.s.	n.s.	0.045	0.076
MW	WW	Science	>	>	>	0.092	0.007	Science Advances	n.s.	n.s.	n.s.	0.064	0.086
WM	WW	Science	n.s.	n.s.	n.s.	-0.053	0.106	Science Advances	n.s.	n.s.	n.s.	0.000	0.995

TABLE S5. **Review sentiment analyses for gendered pairs of authors and reviewers (regular logistic regression).** We use propensity score weighting to control for covariate differences between comparison groups *(53)*, estimated via regular logistic regression. The first letter for a pair represents the corresponding author gender, and the second letter represents the reviewer gender (M=Man, W=Woman). Coefficients represent differences in sentiment score across between groups, while the significance and direction for each pairwise comparison is reported under three conditions: Not corrected (significance threshold = 0.05), the Bonferroni correction (significance threshold = 0.0083), and the Benjamini-Hochberg correction.

Pair 1	Pair 2	Pub.	Not Corrected	Bonferroni	Benjamini-Hochberg	Coef.	p	Pub.	Not Corrected	Bonferroni	Benjamini-Hochberg	Coef.	p
MM	MW	Science	n.s.	n.s.	n.s.	-0.011	0.539	Science Advances	n.s.	n.s.	n.s.	-0.007	0.715
MM	WM	Science	>	>	>	0.100	0.000	Science Advances	>	n.s.	>	0.056	0.025
MM	WW	Science	>	n.s.	>	0.104	0.021	Science Advances	n.s.	n.s.	n.s.	0.050	0.240
MW	WM	Science	>	>	>	0.116	0.000	Science Advances	n.s.	n.s.	n.s.	0.055	0.051
MW	WW	Science	>	n.s.	>	0.097	0.031	Science Advances	n.s.	n.s.	n.s.	0.071	0.104
WM	WW	Science	n.s.	n.s.	n.s.	-0.050	0.143	Science Advances	n.s.	n.s.	n.s.	-0.001	0.988

TABLE S6. **Review sentiment analyses for gendered pairs of authors and reviewers (gradient boosted logistic regression).** We use propensity score weighting to control for covariate differences between comparison groups *(54)*, estimated via gradient boosted logistic regression. The first letter for a pair represents the corresponding author gender, and the second letter represents the reviewer gender (M=Man, W=Woman). Coefficients represent differences in sentiment score across between groups, while the significance and direction for each pairwise comparison is reported under three conditions: Not corrected (significance threshold = 0.05), the Bonferroni correction (significance threshold = 0.0083), and the Benjamini-Hochberg correction.

corresponding authors at European institutions had one of these 150 Chinese surnames.

We then used multivariate regressions to model the manuscript evaluation outcomes for two subpopulations: (a) the manuscripts where the corresponding author had a Chinese last name from either a China-based or the U.S. and Canada-based affiliation, to isolate the location effect; and (b) manuscripts where the corresponding author was affiliated with an institution based in the U.S. and Canada, to isolate the effect of having a Chinese surname.

Fig. S8 shows the results of these analyses. We find that among corresponding authors in the US and Canada, having a Chinese surname is statistically significantly associated with poorer outcomes at every stage of the review process at both journals. Among authors with Chinese surnames, affiliation with a Chinese rather than US or Canadian institution is associated with poorer outcomes as well, in most stages at both journals, except for the sentiment or acceptance stages at *Science*. Informal interviews with all 25 *Science* editors, and 10 *Science Advances* Deputy Editors, over the course of Jan–Oct 2025 suggest that many manuscripts from Chinese institutions describe narrow contributions or are of low quality, consistent with Chinese governmental incentives driving higher submission rates during the time period of the study *(37)*.

Alphabetically, the 150 most common lowercased surnames from authors affiliated with Chinese institutions in submissions to *Science* and *Science Advances* are:

an, bai, bao, bi, bian, bu, cai, cao, chai, chang, chen, cheng, chu, cong, cui, dai, deng, ding, dong, du, duan, fan, fang, feng, fu, gan, gao, ge, geng, gong, gu, guan, guo, han, hao, he, hong, hou, hu, hua, huang, ji, jia, jiang, jiao, jin, jing, ju, kang, ke, kong, lai, lan, lee, lei, li, liang, liao, lin, ling, liu, long, lou, lu, luo, lv, ma, mao, mei, meng, miao, mo, mu, ni, nie, ning, niu, ouyang, pan, pang, pei, peng, piao, pu, qi, qian, qiao, qin, qiu, qu, rao, ren, rong, ruan, shan, shang, shao, shen, sheng, shi, shu, si, song, su, sun, tan, tang, tao, tian, tong, tu, wan, wang, wei, wen, weng, wu, xi, xia, xiang, xiao, xie, xin, xing, xiong, xu, xue, yan, yang, yao, ye, yi, yin, ying, you, yu, yuan, yue, zeng, zhai, zhan, zhang, zhao, zheng, zhong, zhou, zhu, zhuang, zou, and zuo.

Appendix S8: Power analysis for RCTs at *Science*

Double-anonymous randomized controlled trial (RCT) experiments *(21, 40, 57)* would be necessary to assess the causal impact of editors, advisors, and/or reviewers being able to observe variables associated with the identities of the authors, vs. not seeing them. Anonymization does not necessarily have to be complete, e.g., institutional affiliations could be anonymized or not, names could be removed or not, the number of authors could be con-

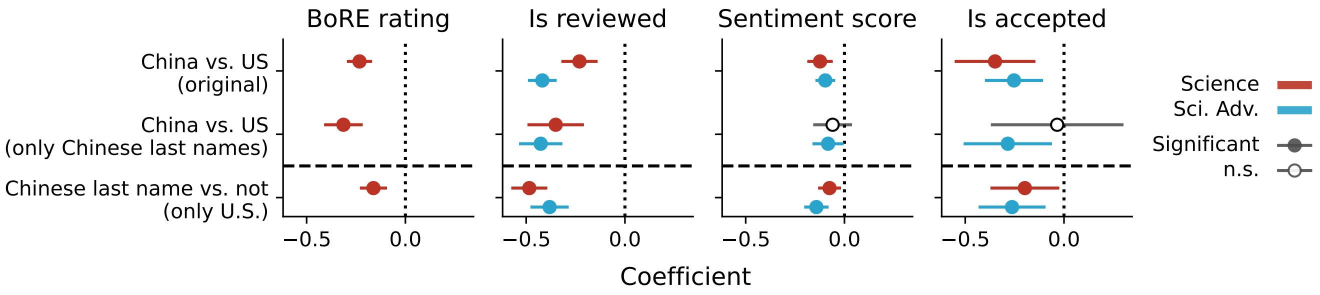

FIG. S8. **Chinese name analysis.** Logistic regression coefficients in three scenarios, comparing *Science* (red) and *Science Advances* (blue) at each stage where applicable. First, comparing China vs. US + Canada. Second, an identical analysis applied to a data set where only manuscripts with US + Canada and Chinese corresponding authors with Chinese last names are included (second row). Third, an analysis restricted to US + Canada corresponding authors, comparing those with Chinese last names vs. not (last row, below dashed line). We control for the same coefficients in the BoRE analysis as in Table S7, for the "Is reviewed" stage as in Table S8, for the sentiment score as in Table S10, and for the "Is accepted" stage as in Table S12. Statistically significant results are shown with a filled-in marker, and non-significant results are shown with a hollow white marker ($\alpha = 0.05$). Error bars display 95% CIs. BoRE rating results only show Science because Science Advances does not have a BoRE.

cealed, etc. The multi-step structure of the evaluation process at *Science* provides many opportunities to intervene via an RCT. However, a key question in undertaking such an experiment is how many manuscripts would need to be included in such a trial in order to assess whether the observed correlations (Fig. 4) are significant.

In such experiments, the BoRE may be a particularly fruitful location for anonymization, as anonymizing manuscripts to editors poses other problems, e.g., editors may need to know author identities in order to manage conflicts of interest or ensuring compliance with certain journal policies. Intervening at this early step, during editorial review, may have a substantial downstream effect on which manuscripts are passed on to peer review.

Assuming that *Science* sends roughly 10,000 manuscripts to the BoRE each year (Fig. 1A), a certain portion of those manuscripts would be placed into the treatment condition and anonymized, and the remainder would be processed as normal (without anonymization), as the control condition. Our estimate for the effect size is based on our modeling results for BoRE ratings (Table S7). However, to allow for the possibly unobserved confounders, we take half the coefficients from the regression models to be a more conservative estimate of the true effect size. We then apply a standard power calculation for two samples of different sizes, for a t-test of means, at $\beta = 0.8$ and $\alpha = 0.05$.

We find that with an experiment with 3000 treatment manuscripts and 7000 control manuscripts could detect effects of half the regression coefficients for differences in BoRE ratings (i) between the highest vs. lowest prestige brackets, (ii) between the US + Canada and China, and (iii) between the smallest (1-5 authors) and largest (10+) teams. However, even at this scale (an entire year of submissions at *Science*), such an RCT would not be guaranteed to detect a gender effect for first or corresponding authors, due to the small magnitude of its coefficient.

Outcome: BoRE advisor rating	*Science*		
Variable	Coef (95% CI)	t	p
[Intercept]	5.20 (5.11, 5.29)	118.17	**0.000
Topic 0 (vs. Topic 9)	0.02 (−0.05, 0.10)	0.60	0.546
Topic 1	−0.12 (−0.19, −0.04)	−3.12	**0.002
Topic 2	0.13 (0.07, 0.19)	4.10	**0.000
Topic 3	−0.34 (−0.43, −0.25)	−7.35	**0.000
Topic 4	0.17 (0.08, 0.26)	3.60	**0.000
Topic 5	−0.08 (−0.15, −0.00)	−2.02	*0.043
Topic 6	0.53 (0.46, 0.61)	13.30	**0.000
Topic 7	0.42 (0.35, 0.48)	12.34	**0.000
Topic 8	−0.69 (−0.78, −0.60)	−15.78	**0.000
Woman First Author (vs. man)	−0.07 (−0.10, −0.03)	−3.68	**0.000
Woman Deputy Editor (vs. man)	−0.18 (−0.22, −0.15)	−9.88	**0.000
10+ authors	0.22 (0.18, 0.26)	10.97	**0.000
1-5 authors	−0.32 (−0.36, −0.28)	−15.44	**0.000
Prestige bins 1-2 (vs. unranked)	0.73 (0.66, 0.79)	22.09	**0.000
Prestige bins 3-4	0.48 (0.42, 0.55)	14.49	**0.000
Prestige bins 5-6	0.32 (0.26, 0.39)	9.92	**0.000
Prestige bins 7-10	0.11 (0.05, 0.18)	3.49	**0.000
CA Nationality China (vs. Other)	−0.23 (−0.30, −0.17)	−7.16	**0.000
CA Nationality Europe	0.26 (0.21, 0.31)	9.89	**0.000
CA Nationality Unknown	0.23 (0.16, 0.31)	6.34	**0.000
CA Nationality US + Canada	0.22 (0.16, 0.27)	8.02	**0.000
Woman Corresponding Author (vs. man)	−0.09 (−0.13, −0.05)	−4.38	**0.000
Woman BoRE advisor (vs. man)	−0.07 (−0.10, −0.03)	−3.45	**0.001
$N = 65048$; $R^2 = 0.06$; * $p < 0.05$, ** $p < 0.005$			

TABLE S7. Linear regression coefficients predicting BoRE advisor rating. Only *Science* coefficients are shown because *Science Advances* does not use the BoRE system. Intercepts for categorical covariates are shown in parentheses. CA = Corresponding Author.

Outcome: Manuscript sent to review	*Science*			*Science Advances*		
Variable	Coef (95% CI)	t	p	Coef (95% CI)	t	p
[Intercept]	−2.46 (−2.58, −2.34)	−40.26	**0.000	−1.48 (−1.61, −1.35)	−22.83	**0.000
Topic 0 (vs. Topic 9)	0.18 (0.08, 0.28)	3.51	**0.000	0.28 (0.16, 0.41)	4.48	**0.000
Topic 1	0.38 (0.29, 0.48)	8.10	**0.000	0.16 (0.05, 0.27)	2.97	**0.003
Topic 2	0.00 (−0.08, 0.09)	0.03	0.976	−0.08 (−0.18, 0.03)	−1.46	0.145
Topic 3	−0.57 (−0.69, −0.45)	−9.24	**0.000	−0.38 (−0.51, −0.25)	−5.56	**0.000
Topic 4	0.08 (−0.03, 0.19)	1.41	0.158	0.22 (0.10, 0.34)	3.58	**0.000
Topic 5	−0.30 (−0.41, −0.19)	−5.28	**0.000	0.29 (0.18, 0.39)	5.46	**0.000
Topic 6	0.17 (0.07, 0.27)	3.28	**0.001	0.69 (0.57, 0.81)	11.29	**0.000
Topic 7	0.16 (0.07, 0.25)	3.44	**0.001	0.09 (−0.02, 0.21)	1.65	0.098
Topic 8	0.28 (0.17, 0.38)	5.21	**0.000	0.13 (0.01, 0.24)	2.17	*0.030
Woman First Author (vs. man)	−0.08 (−0.13, −0.03)	−3.44	**0.001	−0.02 (−0.07, 0.03)	−0.85	0.398
Woman Deputy Editor (vs. man)	0.09 (0.04, 0.14)	3.60	**0.000	−0.34 (−0.40, −0.28)	−11.58	**0.000
10+ authors	0.44 (0.39, 0.49)	16.86	**0.000	0.11 (0.05, 0.16)	3.87	**0.000
1-5 authors	−0.50 (−0.56, −0.45)	−17.96	**0.000	−0.22 (−0.28, −0.16)	−7.61	**0.000
Prestige bins 1-2 (vs. unranked)	1.10 (1.02, 1.18)	26.31	**0.000	0.85 (0.76, 0.95)	17.53	**0.000
Prestige bins 3-4	0.65 (0.57, 0.73)	15.15	**0.000	0.58 (0.49, 0.66)	12.66	**0.000
Prestige bins 5-6	0.39 (0.30, 0.47)	9.11	**0.000	0.43 (0.35, 0.52)	10.18	**0.000
Prestige bins 7-10	0.06 (−0.02, 0.15)	1.42	0.155	0.16 (0.07, 0.24)	3.70	**0.000
CA Nationality China (vs. Other)	−0.23 (−0.32, −0.14)	−4.91	**0.000	−0.42 (−0.49, −0.35)	−11.26	**0.000
CA Nationality Europe	0.50 (0.43, 0.57)	13.36	**0.000	0.34 (0.27, 0.41)	9.36	**0.000
CA Nationality Unknown	0.56 (0.46, 0.65)	11.51	**0.000	0.17 (0.07, 0.27)	3.26	**0.001
CA Nationality US + Canada	0.49 (0.41, 0.56)	12.82	**0.000	0.26 (0.19, 0.33)	7.01	**0.000
Woman Corresponding Author (vs. man)	−0.08 (−0.13, −0.02)	−2.84	**0.005	−0.08 (−0.13, −0.02)	−2.66	*0.008
	$N = 68047$<br>pseudo $R^2 = 0.08$			$N = 42256$<br>pseudo $R^2 = 0.04$		
* $p < 0.05$, ** $p < 0.005$						

TABLE S8. **Logistic regression coefficients predicting whether a manuscript is sent to review.** Intercepts for categorical covariates are shown in parentheses. CA = Corresponding Author.

Outcome: Manuscript sent to review	*Science*		
Variable	Coef (95% CI)	t	p
[Intercept]	−6.09 (−6.28, −5.89)	−60.67	**0.000
BoRE rating	0.65 (0.64, 0.67)	84.82	**0.000
BoRE Confidence	0.03 (0.00, 0.05)	2.33	*0.020
Woman BoRE advisor (vs. man)	0.15 (0.10, 0.20)	5.94	**0.000
Topic 0 (vs. Topic 9)	0.17 (0.05, 0.29)	2.84	**0.005
Topic 1	0.66 (0.55, 0.77)	11.56	**0.000
Topic 2	−0.08 (−0.18, 0.02)	−1.52	0.128
Topic 3	0.05 (−0.10, 0.20)	0.69	0.489
Topic 4	0.26 (0.13, 0.40)	3.78	**0.000
Topic 5	−0.19 (−0.32, −0.06)	−2.79	*0.005
Topic 6	0.24 (0.11, 0.36)	3.78	**0.000
Topic 7	−0.11 (−0.22, −0.00)	−2.02	*0.043
Topic 8	0.61 (0.48, 0.74)	8.96	**0.000
Woman First Author (vs. man)	−0.07 (−0.12, −0.01)	−2.32	*0.020
Woman Deputy Editor (vs. man)	0.19 (0.13, 0.25)	6.27	**0.000
10+ authors	0.36 (0.29, 0.42)	11.11	**0.000
1-5 authors	−0.22 (−0.29, −0.15)	−6.31	**0.000
Prestige bins 1-2 (vs. unranked)	0.55 (0.44, 0.65)	10.52	**0.000
Prestige bins 3-4	0.21 (0.11, 0.31)	3.96	**0.000
Prestige bins 5-6	0.03 (−0.07, 0.13)	0.59	0.556
Prestige bins 7-10	−0.15 (−0.25, −0.04)	−2.75	*0.006
CA Nationality China (vs. Other)	−0.08 (−0.19, 0.04)	−1.34	0.180
CA Nationality Europe	0.35 (0.26, 0.44)	7.76	**0.000
CA Nationality Unknown	0.35 (0.23, 0.47)	5.74	**0.000
CA Nationality US + Canada	0.36 (0.27, 0.45)	7.81	**0.000
Woman Corresponding Author (vs. man)	−0.03 (−0.10, 0.04)	−0.85	0.394
$N = 65048$; $R^2 = 0.22$; * $p < 0.05$, ** $p < 0.005$			

TABLE S9. **Logistic regression coefficients predicting whether a manuscript is sent to review, including controls for BoRE advisor ratings.** Intercepts for categorical covariates are shown in parentheses. CA = Corresponding Author.

Outcome: Review sentiment	*Science*			*Science Advances*		
Variable	Coef (95% CI)	t	p	Coef (95% CI)	t	p
[Intercept]	−0.05 (−0.13, 0.03)	−1.13	0.258	−0.11 (−0.20, −0.03)	−2.61	*0.009
Topic 0 (vs. Topic 9)	0.12 (0.06, 0.19)	3.82	**0.000	0.19 (0.11, 0.27)	4.70	**0.000
Topic 1	−0.08 (−0.14, −0.02)	−2.73	*0.006	−0.08 (−0.15, −0.01)	−2.29	*0.022
Topic 2	−0.12 (−0.17, −0.07)	−4.50	**0.000	−0.03 (−0.10, 0.03)	−0.96	0.337
Topic 3	−0.13 (−0.22, −0.05)	−3.26	**0.001	−0.02 (−0.11, 0.07)	−0.39	0.694
Topic 4	−0.02 (−0.10, 0.05)	−0.63	0.528	−0.01 (−0.09, 0.06)	−0.31	0.758
Topic 5	0.05 (−0.02, 0.13)	1.46	0.143	−0.04 (−0.11, 0.03)	−1.19	0.236
Topic 6	−0.01 (−0.08, 0.05)	−0.33	0.740	0.04 (−0.03, 0.11)	1.05	0.294
Topic 7	0.07 (0.02, 0.13)	2.63	*0.009	0.01 (−0.06, 0.08)	0.34	0.731
Topic 8	−0.05 (−0.11, 0.02)	−1.36	0.175	−0.11 (−0.19, −0.03)	−2.77	*0.006
Woman First Author (vs. man)	−0.00 (−0.03, 0.03)	−0.05	0.961	−0.00 (−0.04, 0.03)	−0.23	0.815
Woman Deputy Editor (vs. man)	0.01 (−0.02, 0.04)	0.74	0.460	0.07 (0.03, 0.11)	3.60	**0.000
Prestige bins 1-2 (vs. unranked)	0.14 (0.09, 0.20)	5.07	**0.000	0.14 (0.08, 0.20)	4.57	**0.000
Prestige bins 3-4	0.09 (0.04, 0.15)	3.27	**0.001	0.09 (0.03, 0.15)	3.07	**0.002
Prestige bins 5-6	0.02 (−0.04, 0.08)	0.71	0.475	0.04 (−0.01, 0.10)	1.51	0.131
Prestige bins 7-10	0.02 (−0.04, 0.08)	0.58	0.564	0.02 (−0.03, 0.08)	0.88	0.381
Woman Corresponding Author (vs. man)	−0.07 (−0.10, −0.03)	−3.82	**0.000	−0.04 (−0.08, −0.00)	−2.23	*0.026
CA Nationality China (vs. Other)	−0.12 (−0.19, −0.06)	−3.76	**0.000	−0.10 (−0.15, −0.05)	−3.81	**0.000
CA Nationality Europe	0.09 (0.04, 0.14)	3.59	**0.000	0.08 (0.04, 0.13)	3.60	**0.000
CA Nationality Unknown	0.08 (0.02, 0.14)	2.48	*0.013	0.10 (0.03, 0.17)	2.84	**0.005
CA Nationality US + Canada	0.05 (0.00, 0.10)	2.02	*0.043	0.10 (0.05, 0.14)	4.00	**0.000
10+ authors	−0.02 (−0.06, 0.01)	−1.44	0.149	0.05 (0.01, 0.08)	2.54	*0.011
1-5 authors	−0.07 (−0.11, −0.03)	−3.83	**0.000	−0.06 (−0.09, −0.02)	−2.93	**0.003
Woman Reviewer (vs. man)	0.03 (−0.00, 0.06)	1.75	0.080	0.00 (−0.03, 0.04)	0.13	0.896
			$N = 23532$			$N = 20965$
			$R^2 = 0.02$			$R^2 = 0.02$
* $p < 0.05$, ** $p < 0.005$						

TABLE S10. **Linear regression coefficients predicting review sentiment.** Intercepts for categorical covariates are shown in parentheses. CA = Corresponding Author.

Outcome: Accepted after review	*Science*			*Science Advances*		
Variable	Coef (95% CI)	t	p	Coef (95% CI)	t	p
[Intercept]	−0.94 (−1.19, −0.68)	−7.08	**0.000	−0.59 (−0.84, −0.34)	−4.58	**0.000
Topic 0 (vs. Topic 9)	0.14 (−0.05, 0.33)	1.43	0.153	0.20 (−0.02, 0.42)	1.81	0.070
Topic 1	0.47 (0.29, 0.64)	5.20	**0.000	0.28 (0.09, 0.47)	2.94	**0.003
Topic 2	−0.26 (−0.42, −0.09)	−3.01	**0.003	0.31 (0.13, 0.50)	3.32	**0.001
Topic 3	−0.33 (−0.60, −0.07)	−2.46	*0.014	−0.33 (−0.58, −0.07)	−2.52	*0.012
Topic 4	−0.03 (−0.26, 0.20)	−0.27	0.787	−0.08 (−0.29, 0.14)	−0.71	0.479
Topic 5	0.43 (0.21, 0.64)	3.90	**0.000	0.23 (0.05, 0.41)	2.49	*0.013
Topic 6	0.64 (0.45, 0.84)	6.48	**0.000	−0.15 (−0.36, 0.06)	−1.41	0.159
Topic 7	0.62 (0.45, 0.78)	7.22	**0.000	0.23 (0.03, 0.42)	2.24	*0.025
Topic 8	0.43 (0.23, 0.63)	4.22	**0.000	−0.06 (−0.27, 0.15)	−0.58	0.564
Woman First Author (vs. man)	−0.08 (−0.17, 0.01)	−1.80	0.072	0.00 (−0.09, 0.09)	0.02	0.986
Woman Deputy Editor (vs. man)	0.03 (−0.07, 0.12)	0.55	0.584	−0.00 (−0.11, 0.10)	−0.09	0.926
10+ authors	0.25 (0.16, 0.34)	5.27	**0.000	0.14 (0.04, 0.24)	2.89	**0.004
1-5 authors	−0.21 (−0.32, −0.10)	−3.85	**0.000	−0.30 (−0.40, −0.20)	−5.89	**0.000
Prestige bins 1-2 (vs. unranked)	0.31 (0.15, 0.47)	3.84	**0.000	0.50 (0.33, 0.66)	5.93	**0.000
Prestige bins 3-4	0.20 (0.04, 0.36)	2.39	*0.017	0.36 (0.20, 0.51)	4.42	**0.000
Prestige bins 5-6	−0.02 (−0.18, 0.15)	−0.19	0.850	0.17 (0.01, 0.32)	2.15	*0.032
Prestige bins 7-10	−0.08 (−0.26, 0.09)	−0.96	0.338	0.01 (−0.14, 0.16)	0.15	0.884
CA Nationality China (vs. Other)	−0.40 (−0.59, −0.20)	−4.01	**0.000	−0.28 (−0.42, −0.14)	−4.02	**0.000
CA Nationality Europe	0.18 (0.04, 0.33)	2.48	*0.013	0.20 (0.08, 0.33)	3.16	**0.002
CA Nationality Unknown	0.20 (0.02, 0.38)	2.14	*0.032	0.13 (−0.05, 0.32)	1.44	0.151
CA Nationality US + Canada	0.17 (0.02, 0.32)	2.24	*0.025	0.24 (0.11, 0.37)	3.68	**0.000
Woman Corresponding Author (vs. man)	−0.13 (−0.24, −0.03)	−2.56	*0.011	−0.15 (−0.25, −0.05)	−2.97	**0.003
Woman Reviewer (vs. man)	−0.05 (−0.11, 0.02)	−1.45	0.148	−0.06 (−0.14, 0.01)	−1.81	0.070
Reviewer prestige 1-2 (vs. unranked)	−0.21 (−0.31, −0.10)	−3.81	**0.000	−0.05 (−0.16, 0.07)	−0.82	0.413
Reviewer prestige 3-4	−0.18 (−0.29, −0.07)	−3.26	**0.001	−0.10 (−0.21, 0.02)	−1.64	0.101
Reviewer prestige 5-6	−0.15 (−0.26, −0.04)	−2.67	*0.008	−0.05 (−0.17, 0.06)	−0.88	0.381
Reviewer prestige 7-10	−0.07 (−0.19, 0.04)	−1.28	0.202	−0.02 (−0.13, 0.10)	−0.28	0.780
			$N = 23532$			$N = 20965$
			pseudo $R^2 = 0.03$			pseudo $R^2 = 0.03$
* $p < 0.05$, ** $p < 0.005$						

TABLE S11. **Logistic regression coefficients predicting whether a manuscript is accepted after being sent to review.** Intercepts for categorical covariates are shown in parentheses. CA = Corresponding Author.

Outcome: Accepted after review	*Science*			*Science Advances*		
Variable	Coef (95% CI)	t	p	Coef (95% CI)	t	p
[Intercept]	−0.84 (−1.14, −0.55)	−5.63	**0.000	−0.88 (−1.26, −0.50)	−4.52	**0.000
Review Sentiment	0.28 (0.23, 0.33)	11.33	**0.000	0.31 (0.26, 0.36)	11.54	**0.000
Review: Excellent and exciting	1.07 (0.94, 1.20)	16.15	**0.000	1.58 (1.30, 1.86)	10.96	**0.000
Review: Above average	−0.19 (−0.32, −0.06)	−2.95	**0.003	0.71 (0.43, 0.98)	5.02	**0.000
Review: Average	−1.16 (−1.35, −0.96)	−11.50	**0.000	−0.57 (−0.86, −0.29)	−3.93	**0.000
Review: Mediocre or poor	−1.72 (−1.89, −1.56)	−20.21	**0.000	−1.32 (−1.61, −1.03)	−8.87	**0.000
Review: Too specialized	−1.20 (−1.37, −1.02)	−13.35	**0.000	−1.05 (−1.37, −0.73)	−6.42	**0.000
Length	−0.04 (−0.08, 0.01)	−1.54	0.124	−0.08 (−0.13, −0.03)	−3.24	**0.001
Topic 0 (vs. Topic 9)	0.08 (−0.13, 0.28)	0.73	0.468	0.03 (−0.20, 0.26)	0.25	0.803
Topic 1	0.51 (0.33, 0.69)	5.53	**0.000	0.32 (0.12, 0.53)	3.12	**0.002
Topic 2	−0.22 (−0.40, −0.05)	−2.50	*0.013	0.41 (0.22, 0.61)	4.12	**0.000
Topic 3	−0.26 (−0.53, 0.02)	−1.84	0.066	−0.28 (−0.56, −0.01)	−2.02	*0.043
Topic 4	0.05 (−0.19, 0.28)	0.37	0.708	−0.03 (−0.26, 0.20)	−0.25	0.806
Topic 5	0.43 (0.20, 0.65)	3.74	**0.000	0.22 (0.03, 0.42)	2.23	*0.026
Topic 6	0.64 (0.44, 0.85)	6.17	**0.000	−0.28 (−0.50, −0.06)	−2.45	*0.014
Topic 7	0.52 (0.34, 0.69)	5.83	**0.000	0.19 (−0.02, 0.40)	1.80	0.072
Topic 8	0.41 (0.21, 0.62)	3.91	**0.000	−0.15 (−0.38, 0.07)	−1.34	0.181
Woman First Author (vs. man)	−0.04 (−0.13, 0.05)	−0.92	0.358	0.00 (−0.09, 0.10)	0.05	0.958
Woman Deputy Editor (vs. man)	−0.00 (−0.10, 0.09)	−0.08	0.934	−0.07 (−0.18, 0.04)	−1.29	0.198
10+ authors	0.26 (0.16, 0.36)	5.18	**0.000	0.12 (0.01, 0.22)	2.21	*0.027
1-5 authors	−0.21 (−0.32, −0.10)	−3.64	**0.000	−0.29 (−0.40, −0.18)	−5.25	**0.000
Prestige bins 1-2 (vs. unranked)	0.20 (0.03, 0.37)	2.33	*0.020	0.42 (0.24, 0.59)	4.62	**0.000
Prestige bins 3-4	0.11 (−0.06, 0.29)	1.27	0.204	0.35 (0.18, 0.52)	4.04	**0.000
Prestige bins 5-6	−0.06 (−0.24, 0.12)	−0.67	0.501	0.13 (−0.03, 0.30)	1.63	0.103
Prestige bins 7-10	−0.13 (−0.31, 0.06)	−1.36	0.175	−0.03 (−0.19, 0.14)	−0.31	0.754
CA Nationality China (vs. Other)	−0.35 (−0.55, −0.14)	−3.35	**0.001	−0.25 (−0.40, −0.11)	−3.37	**0.001
CA Nationality Europe	0.15 (−0.00, 0.31)	1.93	0.054	0.14 (0.01, 0.28)	2.04	*0.041
CA Nationality Unknown	0.17 (−0.02, 0.37)	1.78	0.076	0.07 (−0.12, 0.26)	0.71	0.476
CA Nationality US + Canada	0.16 (0.00, 0.32)	2.01	*0.044	0.17 (0.03, 0.31)	2.43	*0.015
Woman Corresponding Author (vs. man)	−0.08 (−0.19, 0.03)	−1.45	0.147	−0.12 (−0.23, −0.01)	−2.20	*0.028
Woman Reviewer (vs. man)	−0.02 (−0.10, 0.05)	−0.65	0.514	−0.02 (−0.10, 0.07)	−0.37	0.711
Reviewer prestige 1-2 (vs. unranked)	−0.14 (−0.26, −0.02)	−2.31	*0.021	0.05 (−0.08, 0.18)	0.78	0.437
Reviewer prestige 3-4	−0.22 (−0.34, −0.10)	−3.55	**0.000	−0.07 (−0.20, 0.06)	−1.09	0.275
Reviewer prestige 5-6	−0.15 (−0.27, −0.03)	−2.44	*0.015	−0.04 (−0.17, 0.09)	−0.60	0.547
Reviewer prestige 7-10	−0.09 (−0.22, 0.04)	−1.40	0.162	−0.01 (−0.14, 0.12)	−0.17	0.864
	$N = 23532$<br>pseudo $R^2 = 0.19$			$N = 20965$<br>pseudo $R^2 = 0.22$		
* $p < 0.05$, ** $p < 0.005$						

TABLE S12. **Logistic regression coefficients predicting whether a manuscript is accepted after being sent to review, including controls for review sentiment and evaluation.** Intercepts for categorical covariates are shown in parentheses. CA = Corresponding Author.

	# Uses in Topic	% in Topic		# Uses in Topic	% in Topic
**Topic 0 (N = 7751)**			**Topic 1 (N = 11536)**		
1 marine	225	38.93%	1 spin	954	92.17%
2 genome	221	22.85%	2 perovskite	591	54.57%
3 climate	231	16.08%	3 graphene	579	43.57%
4 sex	141	42.60%	4 superconductivity	289	98.63%
5 social	195	18.17%	5 hall	299	98.03%
6 genomic	177	34.57%	6 semiconductor	252	79.50%
7 biodiversity	170	54.31%	7 charge	369	61.09%
8 ecological	157	56.27%	8 insulator	311	94.53%
9 drosophila	121	30.79%	9 films	301	57.33%
10 wild	155	68.28%	10 room	329	65.93%
**Topic 2 (N = 19569)**			**Topic 3 (N = 7329)**		
1 tumor	1077	84.94%	1 covid	1030	60.95%
2 transcription	725	74.21%	2 social	623	58.06%
3 mice	797	56.89%	3 gender	230	87.12%
4 rna	661	60.20%	4 pandemic	265	74.44%
5 receptor	616	52.12%	5 political	131	91.61%
6 immune	602	64.11%	6 economic	179	64.16%
7 targeting	499	69.89%	7 public	157	83.07%
8 mitochondrial	485	72.60%	8 moral	79	97.53%
9 breast	428	89.54%	9 china	175	22.32%
10 metastasis	299	92.28%	10 academic	78	97.50%
**Topic 4 (N = 6252)**			**Topic 5 (N = 10828)**		
1 climate	1005	69.94%	1 hydrogel	366	93.61%
2 warming	478	78.10%	2 print	392	86.92%
3 china	409	52.17%	3 polymer	397	62.23%
4 soil	360	74.07%	4 nanoparticles	281	38.49%
5 tropical	276	67.65%	5 graphene	273	20.54%
6 ocean	271	35.33%	6 tissue	216	30.95%
7 ice	240	37.44%	7 stretchable	152	88.89%
8 north	195	48.99%	8 microscopy	217	49.21%
9 arctic	171	61.07%	9 surfaces	220	65.67%
10 atlantic	168	60.43%	10 colloidal	121	61.11%
**Topic 6 (N = 7681)**			**Topic 7 (N = 12565)**		
1 earthquake	524	87.92%	1 virus	586	69.02%
2 mantle	359	98.63%	2 infection	511	64.20%
3 ocean	335	43.68%	3 rna	383	34.88%
4 subduction	135	100.00%	4 covid	430	25.44%
5 ice	254	39.63%	5 bacteria	387	72.74%
6 seismic	191	88.02%	6 gut	336	60.87%
7 magma	88	100.00%	7 antibody	251	63.87%
8 crust	100	98.04%	8 proteins	322	48.86%
9 slip	130	78.31%	9 receptor	301	25.47%
10 crustal	79	97.53%	10 viral	312	66.52%
**Topic 8 (N = 8758)**			**Topic 9 (N = 8178)**		
1 catalyst	1008	86.52%	1 neuron	821	65.26%
2 perovskite	468	43.21%	2 cortex	520	94.55%
3 batteries	364	97.59%	3 hippocampus	333	91.23%
4 hydrogen	468	66.20%	4 cortical	366	84.92%
5 graphene	459	34.54%	5 mice	406	28.98%
6 lithium	400	91.32%	6 synaptic	276	78.63%
7 ion	427	56.41%	7 sleep	249	82.45%
8 oxide	329	44.88%	8 cognitive	252	59.15%
9 electrochemical	272	79.30%	9 prefrontal	167	95.98%
10 catalysis	254	79.38%	10 receptor	210	17.77%

TABLE S13. **Top ten distinguishing terms for each Topic10 cluster.** Term Frequency-Inverse Document Frequency (TF–IDF) was used to identify the terms most characteristic of each cluster (see Section S2.). For every topic, the table lists the top ten terms in order of TF-IDF score along with their total number of occurrences within that topic and the percentage of all occurrences of that term that appear in that topic (indicating topic specificity).

REFERENCES

1. L. Bornmann, R. Mutz, H.-D. Daniel, A reliability-generalization study of journal peer reviews: A multilevel meta-analysis of inter-rater reliability and its determinants. *PLOS ONE* **5**, e14331 (2010).

2. D. V. Cicchetti, The reliability of peer review for manuscript and grant submissions: A cross-disciplinary investigation. *Behav. Brain Sci.* **14**, 119–135 (1991).

3. B.-C. Björk, D. Solomon, The publishing delay in scholarly peer-reviewed journals. *J. Informet.* **7**, 914–923 (2013).

4. K. Siler, K. Lee, L. Bero, Measuring the effectiveness of scientific gatekeeping. *Proc. Natl. Acad. Sci. U.S.A.* **112**, 360–365 (2015).

5. C. Greiffenhagen, Checking correctness in mathematical peer review. *Soc. Stud. Sci.* **54**, 184–209 (2024).

6. C. J. Lee, C. R. Sugimoto, G. Zhang, B. Cronin, Bias in peer review. *J. Am. Soc. Inf. Sci. Technol.* **64**, 2–17 (2013).

7. L. Waltman, A review of the literature on citation impact indicators. *J. Informet.* **10**, 365–391 (2016).

8. E. C. McKiernan, L. A. Schimanski, C. M. Nieves, L. Matthias, M. T. Niles, J. P. Alperin, Use of the journal impact factor in academic review, promotion, and tenure evaluations. *eLife* **8**, e47338 (2019).

9. V. A. Traag, Inferring the causal effect of journals on citations. *Quant. Sci. Stud.* **2**, 496–504 (2021).

10. E. S. Reich, Science publishing: The golden club. *Nature* **502**, 291–293 (2013).

11. Springer Nature Limited. Editorial criteria and processes (2024); https://www.nature.com/nature/for-authors/editorial-criteria-and-processes [accessed 18 December 2024].

12. American Sociological Association. American Sociological Review: Journal overview and metrics (2024); https://journals.sagepub.com/overview-metric/ASR [accessed 18 December 2024].

13. Massachusetts Medical Society. About NEJM (2024); https://www.nejm.org/about-nejm/about-nejm [accessed 18 December 2024].

14. E. F. P. Luttmer, Report of the Editor, American Economic Review. AEA Papers and Proceedings **116** (2026), pp. 742–760. https://pubs.aeaweb.org/doi/pdfplus/10.1257/pandp.116.742.

15. Nature publishes too few papers from women researchers—That must change. *Nature* **627**, 7–8 (2024).

16. T. Jefferson, P. Alderson, E. Wager, F. Davidoff, Effects of editorial peer review: A systematic review. *JAMA* **287**, 2784–2786 (2002).

17. M. Baldwin, Scientific autonomy, public accountability, and the rise of "peer review" in the Cold War United States. *Isis* **109**, 538–558 (2018).

18. D. E. Koshland Jr., An editor's quest (II). *Science* **227**, 249–249 (1985).

19. E. S. Darling, Use of double-blind peer review to increase author diversity. *Conserv. Biol.* **29**, 297–299 (2015).

20. A. Tomkins, M. Zhang, W. D. Heavlin, Reviewer bias in single- versus double-blind peer review. *Proc. Natl. Acad. Sci. U.S.A.* **114**, 12708–12713 (2017).

21. A. E. Hultgren, N. M. F. Patras, J. Hicks, Blinding reduces institutional prestige bias during initial review of applications for a young investigator award. *eLife* **13**, e92339 (2024).

22. R. M. Borsuk, L. W. Aarssen, A. E. Budden, J. Koricheva, R. Leimu, T. Tregenza, C. J. Lortie, To name or not to name: The effect of changing author gender on peer review. *Bioscience* **59**, 985–989 (2009).

23. V.A. Traag and L. Waltman. Causal foundations of bias, disparity and fairness. arXiv:2207.13665 [cs.DL] (2022).

24. C. Goldin, *Career and Family: Women's Century-Long Journey toward Equity* (Princeton Univ. Press, 2021).

25. L. Hu. Direct effects. *Phenomenal World* (2020).

26. J. M. Garfunkel, M. H. Ulshen, H. J. Hamrick, E. E. Lawson, Effect of institutional prestige on reviewers' recommendations and editorial decisions. *JAMA* **272**, 137–138 (1994).

27. D. Murray, K. Siler, V. Larivière, W. M. Chan, A. M. Collings, J. Raymond, C. R. Sugimoto, Author-reviewer homophily in peer review. bioRxiv 400515 [Preprint] (2019). https://doi.org/10.1101/400515.

28. M. Helmer, M. Schottdorf, A. Neef, D. Battaglia, Research: Gender bias in scholarly peer review. *eLife* **6**, e21718 (2017).

29. D. Card, S. DellaVigna, P. Funk, N. Iriberri, Are referees and editors in economics gender neutral? *Q. J. Econ.* **135**, 269–327 (2020).

30. C. T. Bergstrom and Kevin Gross. Will anyone review this paper? screening, sorting, and the feedback cycles that imperil peer review. arXiv:2507.10734 [physics.soc-ph] (2025).

31. L. Wu, D. Wang, J. A. Evans, Large teams develop and small teams disrupt science and technology. *Nature* **566**, 378–382 (2019).

32. S. B. Borrelle, J. Ringma, K. L. Law, C. C. Monnahan, L. Lebreton, A. M. Givern, E. Murphy, J. Jambeck, G. H. Leonard, M. A. Hilleary, M. Eriksen, H. P. Possingham, H. De Frond, L. R. Gerber, B. Polidoro, A. Tahir, M. Bernard, N. Mallos, M. Barnes, C. M. Rochman, Predicted growth in plastic waste exceeds efforts to mitigate plastic pollution. *Science* **369**, 1515–1518 (2020).

33. J. C. Venter, M. D. Adams, E. W. Myers, P. W. Li, R. J. Mural, G. G. Sutton, H. O. Smith, M. Yandell, C. A. Evans, R. A. Holt, J. D. Gocayne, P. Amanatides, R. M. Ballew, D. H. Huson,

J. R. Wortman, Q. Zhang, C. D. Kodira, X. H. Zheng, L. Chen, M. Skupski, G. Subramanian, P. D. Thomas, J. Zhang, G. L. G. Miklos, C. Nelson, S. Broder, A. G. Clark, J. Nadeau, V. A. Mc, N. Zinder, A. J. Levine, R. J. Roberts, M. Simon, C. Slayman, M. Hunkapiller, R. Bolanos, A. Delcher, I. Dew, D. Fasulo, M. Flanigan, L. Florea, A. Halpern, S. Hannenhalli, S. Kravitz, S. Levy, C. Mobarry, K. Reinert, K. Remington, J. Abu-Threideh, E. Beasley, K. Biddick, V. Bonazzi, R. Brandon, M. Cargill, I. Chandramouliswaran, R. Charlab, K. Chaturvedi, Z. Deng, V. Di Francesco, P. Dunn, K. Eilbeck, C. Evangelista, A. E. Gabrielian, W. Gan, W. Ge, F. Gong, Z. Gu, P. Guan, T. J. Heiman, M. E. Higgins, R. R. Ji, Z. Ke, K. A. Ketchum, Z. Lai, Y. Lei, Z. Li, J. Li, Y. Liang, X. Lin, F. Lu, G. V. Merkulov, N. Milshina, H. M. Moore, A. K. Naik, V. A. Narayan, B. Neelam, D. Nusskern, D. B. Rusch, S. Salzberg, W. Shao, B. Shue, J. Sun, Z. Wang, A. Wang, X. Wang, J. Wang, M. Wei, R. Wides, C. Xiao, C. Yan, A. Yao, J. Ye, M. Zhan, W. Zhang, H. Zhang, Q. Zhao, L. Zheng, F. Zhong, W. Zhong, S. Zhu, S. Zhao, D. Gilbert, S. Baumhueter, G. Spier, C. Carter, A. Cravchik, T. Woodage, F. Ali, H. An, A. Awe, D. Baldwin, H. Baden, M. Barnstead, I. Barrow, K. Beeson, D. Busam, A. Carver, A. Center, M. L. Cheng, L. Curry, S. Danaher, L. Davenport, R. Desilets, S. Dietz, K. Dodson, L. Doup, S. Ferriera, N. Garg, A. Gluecksmann, B. Hart, J. Haynes, C. Haynes, C. Heiner, S. Hladun, D. Hostin, J. Houck, T. Howland, C. Ibegwam, J. Johnson, F. Kalush, L. Kline, S. Koduru, A. Love, F. Mann, D. May, S. Mc Cawley, T. Mc, I. Mc, M. Moy, L. Moy, B. Murphy, K. Nelson, C. Pfannkoch, E. Pratts, V. Puri, H. Qureshi, M. Reardon, R. Rodriguez, Y. H. Rogers, D. Romblad, B. Ruhfel, R. Scott, C. Sitter, M. Smallwood, E. Stewart, R. Strong, E. Suh, R. Thomas, N. N. Tint, S. Tse, C. Vech, G. Wang, J. Wetter, S. Williams, M. Williams, S. Windsor, E. Winn-Deen, K. Wolfe, J. Zaveri, K. Zaveri, J. F. Abril, R. Guigó, M. J. Campbell, K. V. Sjolander, B. Karlak, A. Kejariwal, H. Mi, B. Lazareva, T. Hatton, A. Narechania, K. Diemer, A. Muruganujan, N. Guo, S. Sato, V. Bafna, S. Istrail, R. Lippert, R. Schwartz, B. Walenz, S. Yooseph, D. Allen, A. Basu, J. Baxendale, L. Blick, M. Caminha, J. Carnes-Stine, P. Caulk, Y. H. Chiang, M. Coyne, C. Dahlke, A. D. Mays, M. Dombroski, M. Donnelly, D. Ely, S. Esparham, C. Fosler, H. Gire, S. Glanowski, K. Glasser, A. Glodek, M. Gorokhov, K. Graham, B. Gropman, M. Harris, J. Heil, S. Henderson, J. Hoover, D. Jennings, C. Jordan, J. Jordan, J. Kasha, L. Kagan, C. Kraft, A. Levitsky, M. Lewis, X. Liu, J. Lopez, D. Ma, W. Majoros, J. Mc, S. Murphy, M. Newman, T. Nguyen, N. Nguyen, M. Nodell, S. Pan, J. Peck, M. Peterson, W. Rowe, R. Sanders, J. Scott, M. Simpson, T. Smith, A. Sprague, T. Stockwell, R. Turner, E. Venter, M. Wang, M. Wen, D.

Wu, M. Wu, A. Xia, A. Zandieh, X. Zhu, The sequence of the human genome. *Science* **291**, 1304–1351 (2001).

34. N. Ellemers, Gender stereotypes. *Annu. Rev. Psychol.* **69**, 275–298 (2018).

35. C. R. Sugimoto, V. Larivière, *Equity for Women in Science: Dismantling Systemic Barriers to Advancement* (Harvard Univ. Press, 2023).

36. N. LaBerge, K. H. Wapman, A. Clauset, D. B. Larremore, Gendered hiring and attrition on the path to parity for academic faculty. *eLife* **13**, RP93755 (2024).

37. W. Quan, B. Chen, F. Shu, Publish or impoverish: An investigation of the monetary reward system of science in China (1999-2016). *Aslib J. Inf. Manag.* **69**, 486–502 (2017).

38. M. Pautasso, C. Pautasso, Peer reviewing interdisciplinary papers. *Eur. Rev.* **18**, 227–237 (2010).

39. M. James, Z. Dumlao, M. Teplitskiy, Geographical diversity of peer reviewers shapes author success. *Proc. Natl. Acad. Sci. U.S.A.* **122**, e2507394122 (2025).

40. K. Okike, K. T. Hug, M. S. Kocher, S. S. Leopold, Single-blind vs double-blind peer review in the setting of author prestige. *JAMA* **316**, 1315–1316 (2016).

41. R. Pells. Are academic or professional editors the best for journals? *Times Higher Education* (2018), https://www.timeshighereducation.com/features/are-academic-or-professional-editors-best-journals [accessed 10 November 2025].

42. eLife. eLife's new model: Changing the way you share your research (2022). https://elifesciences.org/inside-elife/54d63486/elife-s-new-model-changing-the-way-you-share-your-research [accessed 10 November 2025].

43. F1000Research. Peer review—Author, referee and editor information (2025). https://f1000research.com/for-authors/peer-review [accessed 10 November 2025].

44. I. Van Buskirk, A. Clauset, D. B. Larremore. "An open-source cultural consensus approach to name-based gender classification," in *Proceedings of the International AAAI Conference on Web and Social Media* (AAAI, 2023), Vol. 17, pp. 866–877.

45. J. W. Lockhart, M. M. King, C. Munsch, Name-based demographic inference and the unequal distribution of misrecognition. *Nat. Hum. Behav.* **7**, 1084–1095 (2023).

46. K. Hunter Wapman, S. Zhang, A. Clauset, D. B. Larremore, Quantifying hierarchy and dynamics in US faculty hiring and retention. *Nature* **610**, 120–127 (2022).

47. US News & World Report, Best Global Universities (2025). https://usnews.com/education/best-global-universities/search.

48. A. Singh, M. D'Arcy, A. Cohan, D. Downey, S. Feldman, SciRepEval: A multi-format benchmark for scientific document representations, in *Proceedings of the 2023 Conference on Empirical Methods in Natural Language Processing* (Association for Computational Linguistics, 2023), pp. 5548–5566. https://aclanthology.org/2023.emnlp-main.338/.

49. NLP Town. bert-base-multilingual-uncased-sentiment(2020). https://huggingface.co/nlptown/bert-base-multilingual-uncased-sentiment [accessed 24 May 2024].

50. N. Kennard, T. O'Gorman, R. Das, A. Sharma, C. Bagchi, M. Clinton, P. K. Yelugam, H. Zamani, A. M. Callum, "DISAPERE: A dataset for discourse structure in peer review discussions," in *Proceedings of the 2022 Conference of the North American Chapter of the Association for Computational Linguistics: Human Language Technologies* (Association for Computational Linguistics, 2022), pp. 1234–1249.

51. K. Imai, L. Keele, D. Tingley, A general approach to causal mediation analysis. *Psychol. Methods* **15**, 309–334 (2010).

52. D. Tingley, T. Yamamoto, K. Hirose, L. Keele, K. Imai, Mediation: R package for causal mediation analysis (2014).

53. N. Greifer. Weightit: Weighting for covariate balance in observational studies. R package version 1.1.0 (2020).

54. G. Ridgeway, D. F. Mc Caffrey, A. R. Morral, M. Cefalu, L. F. Burgette, J. D. Pane, B. A. Griffin, *Toolkit for Weighting and Analysis of Nonequivalent Groups: A Tutorial for the R TWANG Package* (Rand, 2022).

55. H. Bang, J. M. Robins, Doubly robust estimation in missing data and causal inference models. *Biometrics* **61**, 962–973 (2005).

56. Y. Benjamini, Y. Hochberg, Controlling the false discovery rate: A practical and powerful approach to multiple testing. *J. R. Stat. Soc. B. Methodol.* **57**, 289–300 (1995).

57. R. M. Blank, The effects of double-blind versus single-blind reviewing: Experimental evidence from the American Economic Review. *Am. Econ. Rev.* **81**, 1041–1067 (1991).
